# Positive and negative selection shape the human naive B cell repertoire 

**DOI:** 10.1172/JCI150985

**Published:** 2022-01-18

**Authors:** Jeff W. Chen, Jean-Nicolas Schickel, Nikolaos Tsakiris, Joel Sng, Florent Arbogast, Delphine Bouis, Daniele Parisi, Ruchi Gera, Joshua M. Boeckers, Fabien R. Delmotte, Margaret Veselits, Catharina Schuetz, Eva-Maria Jacobsen, Carsten Posovszky, Ansgar S. Schulz, Klaus Schwarz, Marcus R. Clark, Laurence Menard, Eric Meffre

**Affiliations:** 1 Department of Immunobiology, Yale University, New Haven, Connecticut, USA.; 2 Department of Medicine, Section of Rheumatology, Gwen Knapp Center for Lupus and Immunology Research, University of Chicago, Illinois, USA.; 3 Department of Pediatrics and Adolescent Medicine, Ulm University Medical Center, Ulm, Germany.; 4 Department of Pediatrics, Medical Faculty Carl Gustav Carus, Technische Universität Dresden, Dresden, Germany.; 5 Institute for Transfusion Medicine, University of Ulm, Ulm, Germany.; 6 Institute for Clinical Transfusion Medicine and Immunogenetics Ulm, German Red Cross Blood Service Baden-Württemberg, Hessen, Ulm, Germany.; 7 Section of Rheumatology, Allergy and Clinical Immunology, Yale School of Medicine, New Haven, Connecticut, USA.

**Keywords:** Autoimmunity, Immunology, Tolerance

## Abstract

Although negative selection of developing B cells in the periphery is well described, yet poorly understood, evidence of naive B cell positive selection remains elusive. Using 2 humanized mouse models, we demonstrate that there was strong skewing of the expressed immunoglobulin repertoire upon transit into the peripheral naive B cell pool. This positive selection of expanded naive B cells in humanized mice resembled that observed in healthy human donors and was independent of autologous thymic tissue. In contrast, negative selection of autoreactive B cells required thymus-derived Tregs and MHC class II–restricted self-antigen presentation by B cells. Indeed, both defective MHC class II expression on B cells of patients with rare bare lymphocyte syndrome and prevention of self-antigen presentation via HLA-DM inhibition in humanized mice resulted in the production of autoreactive naive B cells. These latter observations suggest that Tregs repressed autoreactive naive B cells continuously produced by the bone marrow. Thus, a model emerged, in which both positive and negative selection shaped the human naive B cell repertoire and that each process was mediated by fundamentally different molecular and cellular mechanisms.

## Introduction

Developing B cells undergo 2 sequential selection steps, whereby clones expressing self-reactive B cell receptors (BCRs) are purged from the repertoire (1). A central tolerance checkpoint occurs in the bone marrow and removes the vast majority of self-reactive immature B cells that express polyreactive BCRs and antinuclear specificities. A second checkpoint that further reduces the frequency of autoreactive B cells occurs in the periphery at the new emigrant/transitional B cell stage before these cells enter the long-lived mature naive B cell compartment (1, 2). The regulation of central B cell tolerance has been extensively studied in both mice and humans and mainly involves the negative selection of autoreactive immature B cells in the bone marrow through the recognition of self-antigens by BCRs and potentially TLRs (3–8). In contrast, the cellular and molecular mechanisms presiding at the establishment of the peripheral B cell tolerance checkpoint probably involve both negative and positive selection but are not well understood (9–11).

Early studies with engineered monoclonal T and B cells showed that CD4^+^ T cells played a role in peripheral counterselection of autoreactive B cells (12, 13). In addition, transgenic mouse models have revealed BCR-mediated selection of transitional B cell clones in the mature naive follicular B cell compartment (11). To explore mechanisms that shape the mature naive B cell compartment in humans, we used NOD-*scid*-common γ chain–knockout (NSG) mice transplanted with human fetal hematopoietic stem cells (HSCs) with or without a human thymic graft (14–17). Although both models recapitulated normal central B cell tolerance and peripheral positive selection, fetal thymic tissue was required to repress the peripheral accumulation of autoreactive naive B cells. We found that this thymus-dependent repression was mediated by Tregs and required MHC class II–restricted presentation by autoreactive B cells. These data demonstrate an essential and potentially direct role for Tregs in human B cell peripheral tolerance.

## Results

### Peripheral B cell negative selection requires the presence of an autologous thymus.

**Figure 1:**
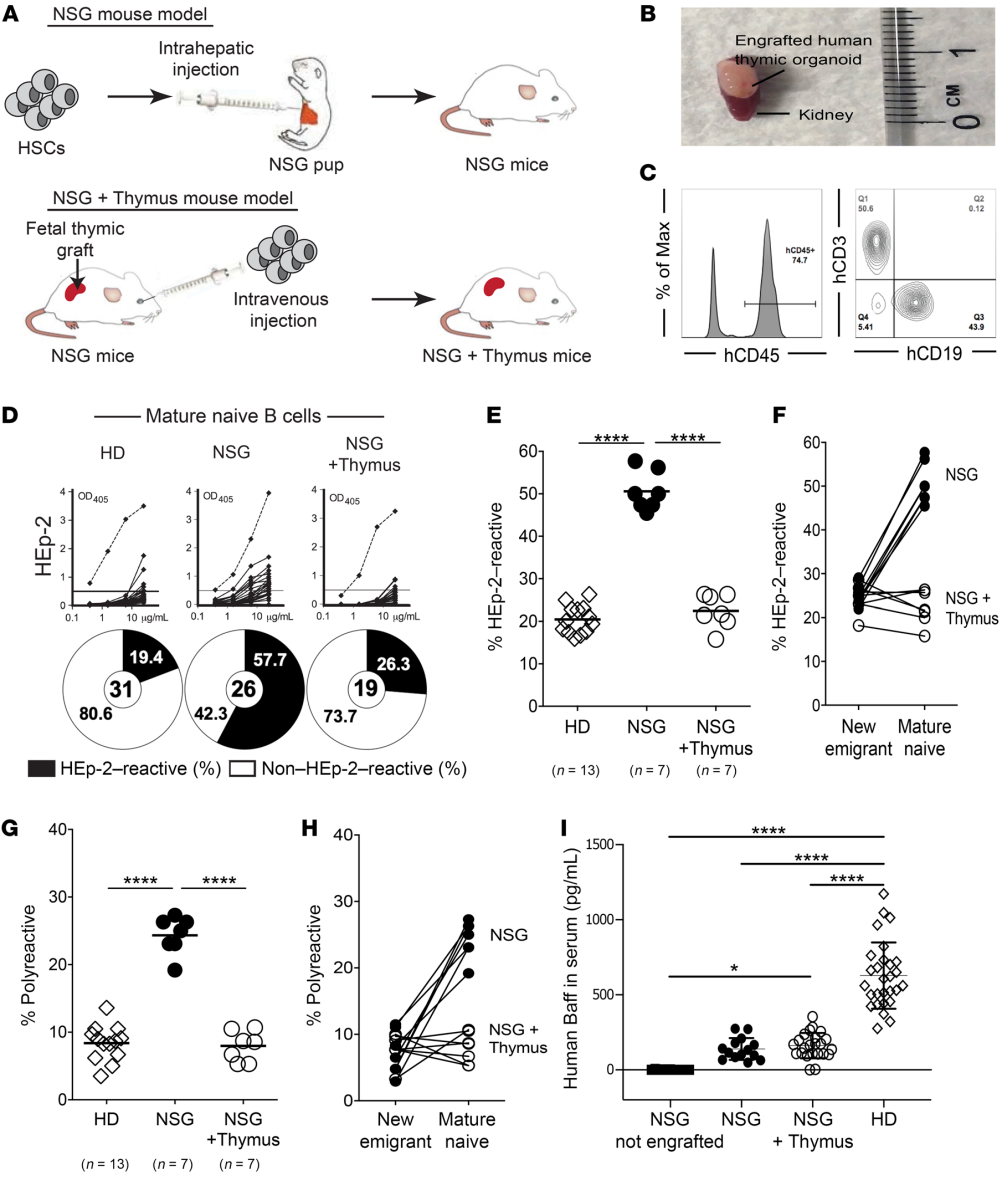
Peripheral selection of autoreactive naive B cells requires the presence of an autologous thymus. (**A**) Schematic diagram depicting the generation of 2 humanized mouse models. CD34^+^ HSCs were injected into the liver of NSG pups (NSG mouse model) or i.v. into adult mice, along with surgical implantation of a piece of autologous thymus under the kidney capsule (NSG + thymus mouse model). (**B**) Representative image of the engrafted human thymic organoid upon sacrifice of the humanized mouse. (**C**) Representative flow cytometric analysis of the frequency of human (h) CD45^+^, CD3^+^, and CD19^+^ cells, reflecting the extent of engraftment in the blood of humanized mice. Max, maximum; Q, quadrant.(**D**) Recombinant Abs cloned from mature naive B cells from HDs (*n =* 13), NSG humanized mice (*n =* 7), and NSG + thymus humanized mice (*n =* 7) were tested by ELISA for anti–HEp-2 cell reactivity. Dotted lines show the ED38 positive control. Horizontal lines show the cutoff OD_405_ for positive reactivity. For each individual or humanized mouse, the frequency of nonreactive (white area) and reactive (black area) clones is summarized in a pie chart below, with the total number of clones tested indicated in the centers. The frequencies of HEp-2–reactive and polyreactive mature naive B cells are summarized in **E** and **G**, respectively. Each symbol represents an individual or humanized mouse. Solid lines show the mean. The frequencies of (**F**) HEp-2–reactive and (**H**) polyreactive B cells and their evolution between the new emigrant/transitional and mature naive B cell stages in NSG and NSG + thymus humanized mice. (**I**) Human BAFF concentrations were measured by ELISA in the sera of nonengrafted NSG mice, NSG humanized mice, NSG + thymus humanized mice, and HDs. *P < 0.05 and ****P < 0.0001, by Mann-Whitney *U* test (**E**, **G**, and **I**).

We investigated the establishment of early B cell tolerance checkpoints in 2 humanized mouse models, both of which relies on the engraftment of human fetal HSCs in NSG immunodeficient mice (Figure 1A). The NSG plus thymus (referred to hereafter as NSG + thymus) humanized mouse model also includes the transplantation of a small fragment of autologous thymic tissue under the mouse kidney capsule and may better support the development of human T cells (Figure 1, A and B, and refs. 18–21). The engrafted human thymic organoid was functional and contained many CD4^+^CD8^+^ double-positive thymocytes, whereas only single-positive CD4^+^ or CD8^+^ T cells were present in the periphery of these mice (Supplemental Figure 1A; supplemental material available online with this article; https://doi.org/10.1172/JCI150985DS1). To standardize the analysis of humanized mice, we studied B cell tolerance when the proportion of human CD45^+^ cells in the blood of both NSG and NSG + thymus humanized mice exceeded 50% of the lymphoid gate and of which approximately half were B cells and 45%–70% were T cells (Figure 1C and Supplemental Figure 1, B and C). NSG and NSG + thymus humanized mice displayed similar numbers of total splenocytes, human B cells, and CD4^+^ T cells, which were lower than counterparts in intact murine spleens, revealing a lymphopenic state in these models as previously reported (Supplemental Figure 1, D and E, and refs. 18–22). The frequencies of naive and memory CD4^+^ and CD8^+^ T cell subsets were not different between healthy human donors (HDs), NSG humanized mice, and NSG + thymus humanized mice (Supplemental Figure 1F). In addition, the humanized mice exhibited no overt signs of T cell activation, as evidenced by a lack of PD-1 induction that usually precedes the development of graft-versus-host disease (data not shown). We next analyzed the repertoire and reactivity of recombinant Abs cloned from single human CD19^+^CD27^–^CD10^+^CD21^–/lo^IgM^hi^ new emigrant/transitional B cells and CD19^+^CD27^–^CD10^–^CD21^+^IgM^+^ mature naive B cells isolated from the spleens of 7 NSG humanized mice and 7 NSG + thymus humanized mice engrafted with donor-matched HSCs isolated from 7 different human fetal samples (1, 14). Despite their human fetal stem cell origin, we found that new emigrant/transitional B cells from both types of humanized mice differed from their fetal counterparts, in that they expressed heavy chains (IgH) with significantly longer complementarity determining regions 3 (CDR3), decreased D7-27 and J_H_2 gene segment usage, and increased positive charge density, features that were associated with Abs expressed by B cells in adults (Supplemental Figure 2, A–E, Supplemental Table 1, and ref. 23). In agreement with the more mature Ig repertoire in humanized mice versus fetuses, new emigrant/transitional B cells in NSG and NSG + thymus humanized mice contained polyreactive and antinuclear reactive clones at low frequencies comparable to those in HDs, whereas fetal counterparts were recently reported to often express polyreactive Abs (Supplemental Figure 2, F–J, and ref. 23). Hence, central B cell tolerance was properly established in both humanized mouse models and independently of a human thymic graft (15).

In contrast, the mature naive B cell compartment of NSG humanized mice contained greater than 2-fold higher proportions of self-reactive (HEp-2–reactive) B cells compared with those in donor-matched NSG + thymus humanized mice or HD controls (*P* < 0.0001; Figure 1, D–F). Similarly, recombinant Abs cloned from mature naive B cells in NSG humanized mice were also significantly more polyreactive than the NSG + thymus mice and HD counterparts (*P* < 0.0001; Figure 1, G and H, and Supplemental Figure 1G). Serum concentrations of mouse and human BAFF, an important B cell survival factor that regulates the number of peripheral B cells and may favor autoreactive B cell survival, were similar between NSG and NSG + thymus humanized mice and therefore do not account for the peripheral accumulation of autoreactive B cells in the absence of a human thymic graft (Figure 1I, Supplemental Figure 1H, and ref. 24). The sequence analysis of heavy chains expressed by mature naive B cells using Adaptive Technologies’ immunoSEQ revealed that the impaired peripheral B cell tolerance checkpoint in NSG humanized mice was associated with a BCR repertoire enriched in shorter IgH CDR3s, as well as with increased JH4 and decreased JH6 gene usage that were characteristic of autoreactivity in fetal human B cells (Supplemental Figure 3, A–D, and ref. 23). In contrast, mature naive B cells from NSG + thymus humanized mice expressed a BCR repertoire similar to that in HDs (Supplemental Figure 3, A–E). Thus, the presence of an autologous human thymic graft in NSG + thymus humanized mice inhibited the peripheral accumulation of autoreactive B cells observed in NSG humanized mice.

### Homeostatic expansion of mature naive B cells does not require an autologous thymus.

**Figure 2:**
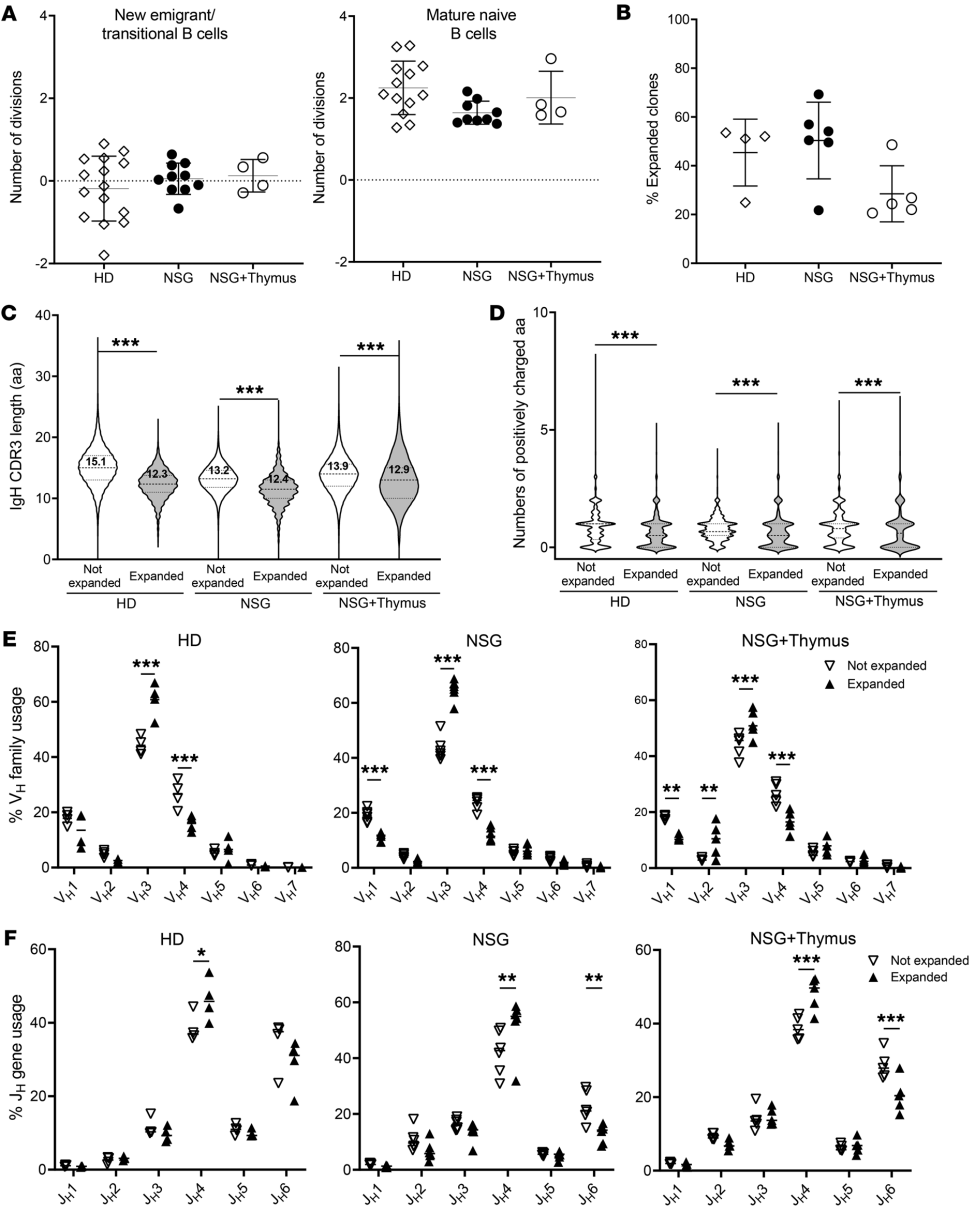
Peripheral positive B cell selection allows the expansion of clones expressing specific BCRs. (**A**) Evaluation of the number of cell divisions that occurred in vivo by KREC analysis of new emigrant/transitional and mature naive B cells isolated from the blood of HDs (*n =* 15) and the spleens of NSG (*n =* 10) or NSG + thymus (*n =* 4) humanized mice. (**B**) Proportions of IgH CDR3s identified more than once (expanded clones) in IgH sequences from mature naive B cells isolated from 4 HDs (146,604 sequences), 6 NSG humanized mice (133,800 sequences), and 5 NSG + thymus (45,223 sequences) humanized mice. Violin plots represent the IgH CDR3 length in amino acids (**C**) and numbers of positively charged amino acids, i.e., arginine, lysine, and histidine in IgH CDR3s (**D**) of nonexpanded versus expanded clones in HDs and NSG and NSG + thymus humanized mice. V_H_ family usage (**E**) and J_H_ gene usage (**F**) of nonexpanded versus expanded mature naive B cells in HDs, NSG humanized mice, and NSG + thymus humanized mice. *P < 0.05, **P < 0.01, ***P < 0.001, by 2-way ANOVA followed by Tukey’s post hoc multiple-comparison test.

Using a quantitative PCR–based assay that measures κ deletion recombination excision circles (KRECs) to obtain an estimate of the number of divisions undergone by a B cell population, we found that mature naive, but not new emigrant/transitional, B cells from HDs underwent an average of 2 divisions in vivo, revealing that some clones were positively selected in the periphery as previously reported (Figure 2A and ref. 25). Mature naive B cells from NSG and NSG + thymus humanized mice also underwent an in vivo homeostatic expansion that was similar to that in HDs, whereas new emigrant/transitional B cells from these humanized mice did not show any sign of proliferation (Figure 2A). Hence, the presence of a human autologous thymic graft was not required for the expansion of some naive B cells in the periphery. In agreement with these observations, the immunoSEQ analysis of the BCR repertoires of mature naive B cells isolated from the blood of 4 HDs and the spleens of 5 NSG and 5 NSG + thymus humanized mice revealed that the proportion of expanded clones assessed by the identification of IgH CDR3s was similar between HDs and humanized mice (Figure 2B). Of note, the expansion of mature naive B cells was limited, since 98.7% ± 0.53% of expanded B cells in HDs, 95.8% ± 2.65% in NSG humanized mice, and 98.3% ± 1.03% in NSG + thymus humanized mice were found 2–4 times (Supplemental Figure 3F). This suggests that most B cells had indeed undergone only 1–2 divisions.

To determine whether the positive selection of expanded B cells in humans involves the amplification of clones with specific BCR specificities, as suggested in mice (10, 11), we compared the BCR repertoires of expanded versus nonexpanded mature naive B cells. We found that expanded mature naive B cells from HDs and humanized mice expressed BCRs with significantly shorter IgH CDR3s containing decreased numbers of positively charged amino acids compared with nonexpanded clones (Figure 2, C and D). In addition, BCRs encoded by VH3 family genes and JH4 gene segments were significantly favored in expanded clones, whereas BCRs encoded by VH1 family genes and JH6 were disfavored, regardless of whether mature naive B cells developed in the HDs or humanized mice (Figure 2, E and F). We conclude that positive selection of expanded mature naive B cells in the periphery occurred independently of the presence of a human autologous thymic graft in humanized mice and resulted in the proliferation of clones expressing a different BCR repertoire compared with nonexpanded clones. Specific antigens encountered in the periphery may therefore regulate the maturation of some naive B cells.

### Tregs prevent the accumulation of autoreactive B cells in the periphery.

**Figure 3:**
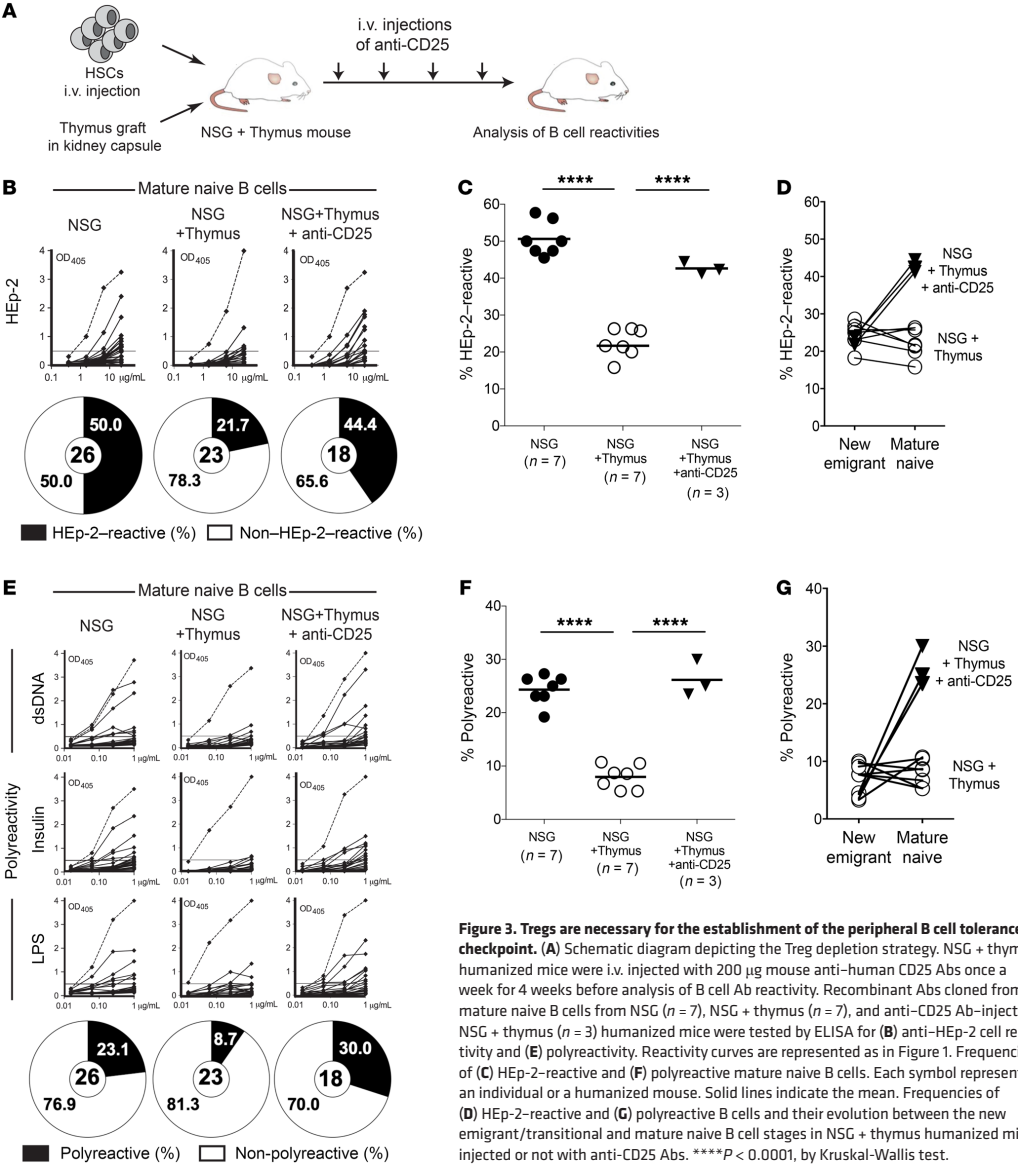
Tregs are necessary for the establishment of the peripheral B cell tolerance checkpoint. (**A**) Schematic diagram depicting the Treg depletion strategy. NSG + thymus humanized mice were i.v. injected with 200 μg mouse anti–human CD25 Abs once a week for 4 weeks before analysis of B cell Ab reactivity. Recombinant Abs cloned from mature naive B cells from NSG (*n =* 7), NSG + thymus (*n =* 7), and anti–CD25 Ab–injected NSG + thymus (*n =* 3) humanized mice were tested by ELISA for (**B**) anti–HEp-2 cell reactivity and (**E**) polyreactivity. Reactivity curves are represented as in Figure 1. Frequencies of (**C**) HEp-2–reactive and (**F**) polyreactive mature naive B cells. Each symbol represents an individual or a humanized mouse. Solid lines indicate the mean. Frequencies of (**D**) HEp-2–reactive and (**G**) polyreactive B cells and their evolution between the new emigrant/transitional and mature naive B cell stages in NSG + thymus humanized mice injected or not with anti-CD25 Abs. ****P < 0.0001, by Kruskal-Wallis test.

We then focused on studying mechanisms of negative selection of autoreactive B cells in the periphery, which previous studies using transgenic murine systems suggested involvement of T cells (12, 13). Other studies of patients with various primary immunodeficiencies pointed to Tregs as potential players controlling the peripheral selection of autoreactive clones that escape central B cell tolerance (6, 26–29). To assess whether Tregs shape the mature naive B cell compartment, we depleted Tregs in well-engrafted NSG + thymus humanized mice by i.v. injection of mouse anti–human CD25 Abs (Figure 3A). To determine the duration of this anti-CD25 regimen, we performed a set of BrdU pulse-labeling experiments to gauge the turnover rates of human lymphocytes in these humanized mice (Supplemental Figure 4A). Both T and B cells had longer lifespans in NSG + thymus humanized mice than in NSG humanized mice, as illustrated by the slower disappearance of BrdU-labeled lymphocytes in the presence of an autologous human thymic graft (Supplemental Figure 4B). As expected, the vast majority of new emigrant/transitional B cells were BrdU labeled within 2 weeks, and nearly all labeled cells were depleted within the following 10 days (Supplemental Figure 4C). In contrast, mature naive B cells showed a slower turnover than did new emigrant B cells, and we therefore estimated their half-life in the humanized mice to be approximately 2 weeks (Supplemental Figure 4C). Thus, we chose a 4-week anti-CD25 injection regimen to allow most clones of the mature naive B cell compartment to develop after Treg depletion (Figure 3A). Of note, splenic Treg/B cell localization appeared similar between the 2 humanized mouse models (Supplemental Figure 4D). The 3 NSG + thymus humanized mice selected for the Treg depletion experiments had typical human immune reconstitution and B and T cell frequencies comparable to those in regular NSG + thymus humanized mice, and well-developed thymic grafts containing CD4^+^CD8^+^ thymocytes (Supplemental Figure 5, A and B and data not shown).

Flow cytometric analysis of blood from NSG + thymus humanized mice injected with anti-CD25 Abs showed that CD4^+^CD25^hi^CD127^lo^FOXP3^+^ Tregs were efficiently depleted within 1 week of the initial Ab injection and remained virtually absent throughout the following weeks before the analysis (Supplemental Figure 5, C and D). Unlike mouse studies in which removal of FOXP3^+^ Tregs led to “catastrophic autoimmunity” (30), the depletion of Tregs by anti-CD25 Abs in these humanized mice did not result in fulminant autoimmune manifestations, as the animals remained asymptomatic, although some T cells acquired an activated phenotype as illustrated by the potentially transient expression of FOXP3 in some CD25^–^ T cells that has been associated with T cell activation in humans and by a modest upregulation of programmed cell death 1 (PD-1) (Supplemental Figure 5, C and E, and refs. 31, 32). This difference may be attributable to decreased innate antigen-presenting cells and human cytokine concentrations in the serum to propagate full-blown inflammation in this particular humanized mouse system compared with regular SPF mice (30, 33). Strikingly, the acute depletion of Tregs in NSG + thymus humanized mice resulted in the expansion of HEp-2–reactive and polyreactive mature naive B cells (Figure 3, B–G). The removal of Tregs by the anti-CD25 regimen did not interfere with central B cell tolerance, since new emigrant/transitional B cells had low proportions of polyreactive and antinuclear clones that were similar to the clones in unmanipulated NSG + thymus humanized mice (Figure 3, D and G, and Supplemental Figure 5, F and G). These observations therefore highlight the specific contribution of Tregs to peripheral B cell negative selection.

### Treg-suppressive function and the appropriate gene signature are associated with a functional peripheral B cell tolerance checkpoint.

**Figure 4:**
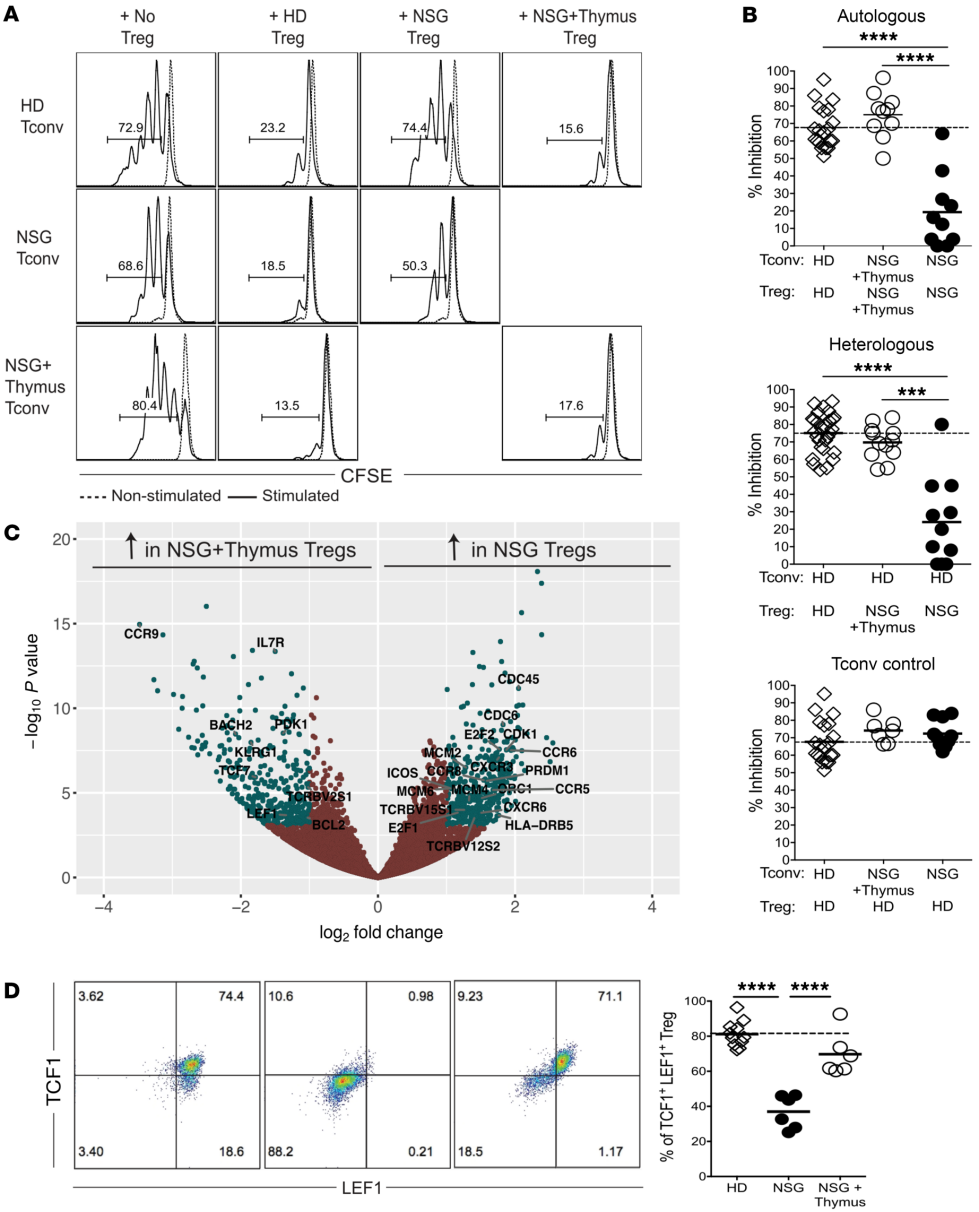
Tregs from NSG humanized mice display defective suppressive capacity and a dysregulated transcriptome and phenotype. (**A**) Representative flow cytometric analysis of Treg-mediated in vitro suppression of autologous and heterologous CFSE-labeled conventional Tconv cells on day 3.5. Dashed line shows nonstimulated Tconv cells. (**B**) Summaries of the suppressive capacity of Tregs from HDs, NSG humanized mice, and NSG + thymus humanized mice in autologous and heterologous settings. The bottom panel shows the percentage of suppression in control settings, where HD Tregs were paired with Tconv cells from HDs, NSG humanized mice, or NSG + thymus humanized mice. (**C**) Volcano plot from the RNA-Seq analysis (*x* axis shows the log_2_ fold change; *y* axis shows –log_10_ adjusted *P* values). Significantly differentially expressed genes are plotted as green dots, and select genes are labeled. The right half of the graph shows genes that were upregulated in NSG Tregs, and the left half shows genes that were upregulated in NSG + thymus Tregs. (**D**) Representative flow cytometric plots and summary of TCF1 and LEF1 expression in Tregs from HDs, NSG, and NSG + thymus humanized mice. ***P < 0.001 and ****P < 0.0001, by Kruskal-Wallis test.

To identify Treg features associated with proper or impaired autoreactive B cell selection in the periphery of NSG versus NSG + thymus humanized mice, respectively, we examined a diverse set of parameters including Treg frequencies, suppressive function, and transcriptomes. Differences in peripheral B cell selection did not result from altered Treg frequencies or FOXP3 or CTLA-4 expression, which were all comparable between these 2 humanized mouse models and higher than those in HDs (Supplemental Figure 6, A–D). In contrast, we found that Tregs from NSG + thymus, but not NSG, humanized mice were capable of suppressing the proliferation of autologous conventional T (Tconv) cells from the same animals or heterologous Tconv cells isolated from the blood of HDs (Figure 4A). Importantly, the proliferation of Tconv cells from NSG and NSG + thymus humanized mice could be inhibited similarly by Tregs from HDs, revealing that the lack of suppression by Tregs from NSG humanized mice was due to an intrinsic functional defect rather than to Tconv cells being refractory to Treg suppression (Figure 4B).

To characterize the molecular signatures associated with effective or impaired Treg-suppressive function, we performed RNA-Seq to analyze the transcriptomes of CD3^+^CD4^+^CD25^hi^CD127^lo^ human Tregs isolated from the spleens of NSG and NSG + thymus humanized mice as well as Tregs from the blood of HDs. We observed stark differences between Tregs from NSG and NSG + thymus humanized mice: Tregs from NSG, but not NSG + thymus, humanized mice or HDs showed upregulated expression of several markers of activated T cells including *CXCR3*, *CCR5*, *ICOS*, and *PRDM1*, which encodes BLIMP-1 (Figure 4, C and D, and Supplemental Figure 6E). Gene ontogeny analysis revealed that, of the approximately 400 genes differentially upregulated in Tregs from NSG humanized mice compared with NSG + thymus humanized mice, 77 genes, including *CDK1* and *E2F1*, belonged to the mitotic cell cycle and favored proliferation, which correlated with increased expression of the proliferation marker Ki67 in Tregs from NSG humanized mice compared with counterpart Tregs in NSG + thymus humanized mice and HDs (Figure 4C and Supplemental Figure 6, E–G). Gene ontogeny analysis also revealed a gene signature promoting an apoptotic process in Tregs from NSG humanized mice, and flow cytometric analysis revealed their low expression of the antiapoptotic factor BCL-2 (Supplemental Figure 6, F and G). In addition, Tregs from NSG humanized mice lacked *CCR7* but showed increased CXCR3 expression compared with Tregs from NSG + thymus humanized mice and HDs (Supplemental Figure 6, E, H, and I). Furthermore, *BACH2*, *LEF1*, and *TCF7*, which encodes the transcription factor TCF1, have all been shown to be important for Treg-suppressive function and were among the genes that were more highly expressed in Tregs from HDs and NSG + thymus humanized mice compared with Tregs from NSG humanized mice (Figure 4, C and D, and refs. 34–36). In summary, Tregs from NSG + thymus humanized mice, in which autoreactive B cells were properly purged from the peripheral repertoire, displayed a transcriptome, phenotype, and suppressive function that were similar to those of HD Tregs, whereas Tregs from NSG humanized mice had a distinct transcriptomic signature that correlated with a defective peripheral B cell tolerance checkpoint.

### Impaired peripheral B cell negative selection in patients with atypical bare lymphocyte syndrome.

**Figure 5:**
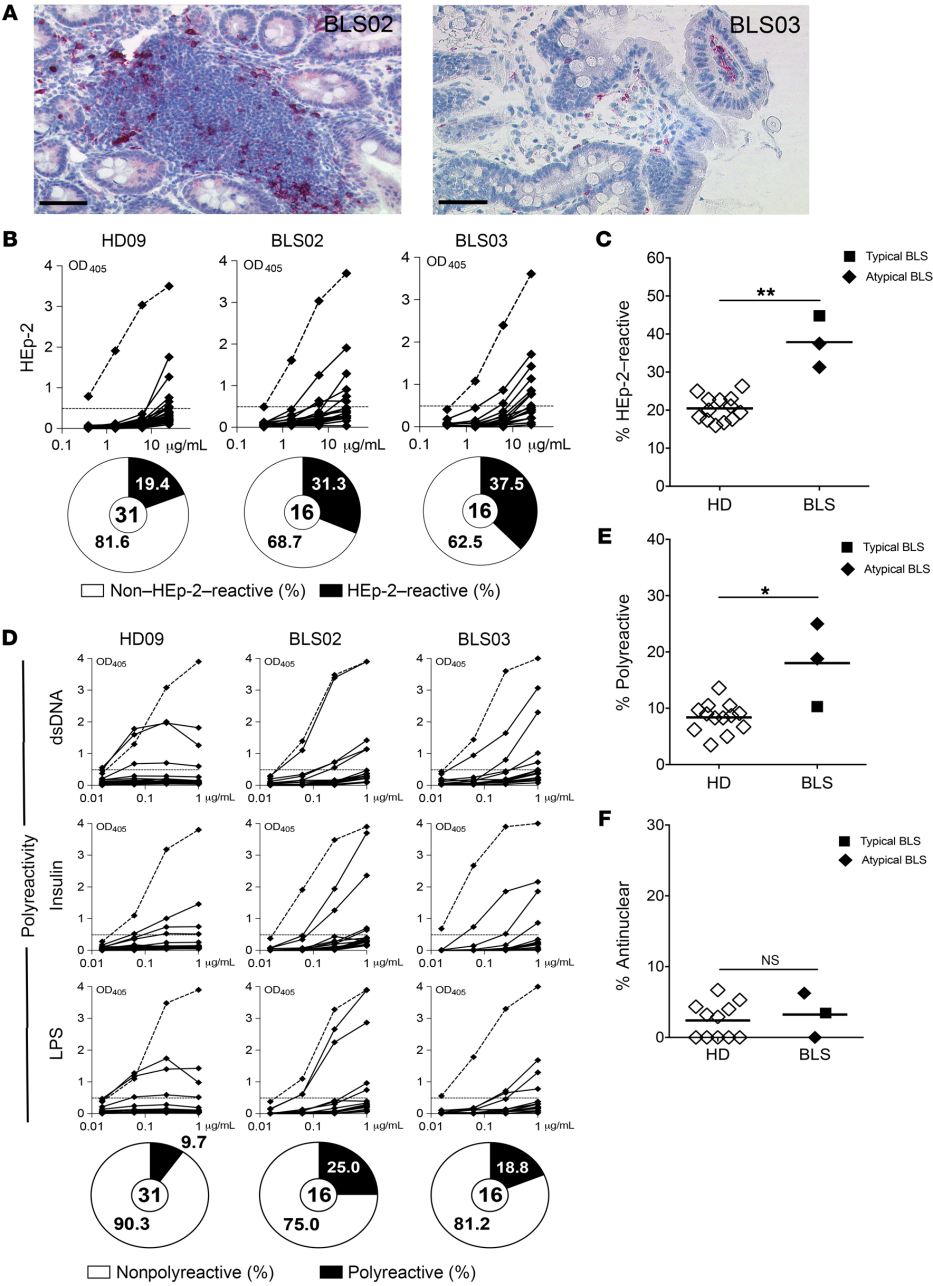
Patients with B cell-selective MHC Class II deficiency harbor elevated proportions of autoreactive mature naive B cells. (**A**) Residual HLA-DR expression was detected in patients BLS02 and BLS03. Formalin-fixed biopsy specimens from the duodenum of patient BLS02 and rectum of patient BLS03 were stained with anti–HLA-DR. Scale bars: 100 μm. (**B**) HEp-2 reactivity and (**D**) polyreactivity of recombinant Abs cloned from single mature naive B cells from a representative HD (HD09) and patients BLS02 and BLS03. Reactivity curves are represented as in Figure 1. Plots showing results for (**C**) HEp-2 reactivity, (**E**) polyreactivity, and (**F**) antinuclear reactivity in the mature naive B cell compartment. Each white diamond represents a HD, each black diamond represents a new patient with atypical BLS, and the black square represents the previously reported patient with typical BLS (40). *P < 0.05 and **P < 0.01, by Mann-Whitney *U* test.

Transcriptomic analyses revealed significant differential expression of several genes encoding specific TCR Vβ segments (*TRBV* genes) in Tregs from NSG humanized mice compared with counterpart Tregs from NSG + thymus humanized mice and HDs (Figure 4C and Supplemental Table 2). An alteration of the TCR repertoire when human thymocytes develop in a murine thymus may contribute to defective autoreactive B cell selection in the periphery. To determine whether T cells can recognize self-antigens presented by MHC class II molecules on autoreactive B cells, we studied 2 patients with unconventional type II bare lymphocyte syndrome (BLS), designated hereafter as patients BLS02 and BLS03, who harbored loss-of-function mutations in *RFXAP* and *RFXANK*, respectively (Supplemental Table 3). Both genes encode for transactivators that bind the X-box motif of MHC class II gene promoters and induce their transcription (37–39). Unlike patients with classical type II BLS, who typically show low (<20%) frequencies of CD4^+^ T cells within the CD3^+^ T cell compartment — including 1 previously studied patient who carried a defective *CIITA* mutation (BLS01; ref. 40) — we found that patients BLS02 and BLS03 had 38.5% and 63.7% CD4^+^ T cells, respectively, among their circulating T cells (Supplemental Figure 7A and ref. 41). These data suggest that some expression of MHC class II molecules on their thymic stroma supported the development of CD4^+^ T cells. Biopsy analysis detected the peripheral expression of MHC class II in these atypical BLS patients through the identification of HLA-DR^+^ cells in the duodenum of patient BLS02 and the rectum of patient BLS03 (Figure 5A). Patient BLS02 was 15 years old at the time of diagnosis and survived more than 10 years prior to HSC transplantation in spite of her defective *RFXAP* gene (Supplemental Table 3). In contrast to the inferred thymic expression and confirmed gastrointestinal expression of MHC class II, we found that freshly isolated B cells from patients BLS02 and BLS03 completely lacked HLA-DR expression at steady state and even after activation by stimulation with diverse agonists including anti-IgM, TLR9 ligand CpG, TLR7 ligand Gardiquimod, and CD40L (Supplemental Figure 7, B–D). Thus, these patients with atypical BLS provided a unique opportunity to evaluate the impact of in vivo B cell–intrinsic MHC class II deficiency on the outcomes of peripheral B cell selection in humans.

Analysis of recombinant Abs cloned from new emigrant/transitional B cells isolated from the blood of patients BLS02 and BLS03 showed low frequencies of polyreactive and antinuclear clones that were similar to the frequencies in HDs, suggesting that MHC class II expression is not necessary for the establishment of central B cell tolerance (Supplemental Figure 7, E and F). In contrast, both atypical BLS patients harbored significant expansions of autoreactive and polyreactive clones in their mature naive B cell compartments, although antinuclear reactivity determined by indirect immunofluorescence remained scarce and comparable to that in healthy individuals (Figure 5, B–F). Hence, MHC-mediated self-peptide presentation by B cells may be required for a functional peripheral B cell tolerance checkpoint.

### B cell self-antigen presentation is required for peripheral B cell negative selection.

**Figure 6:**
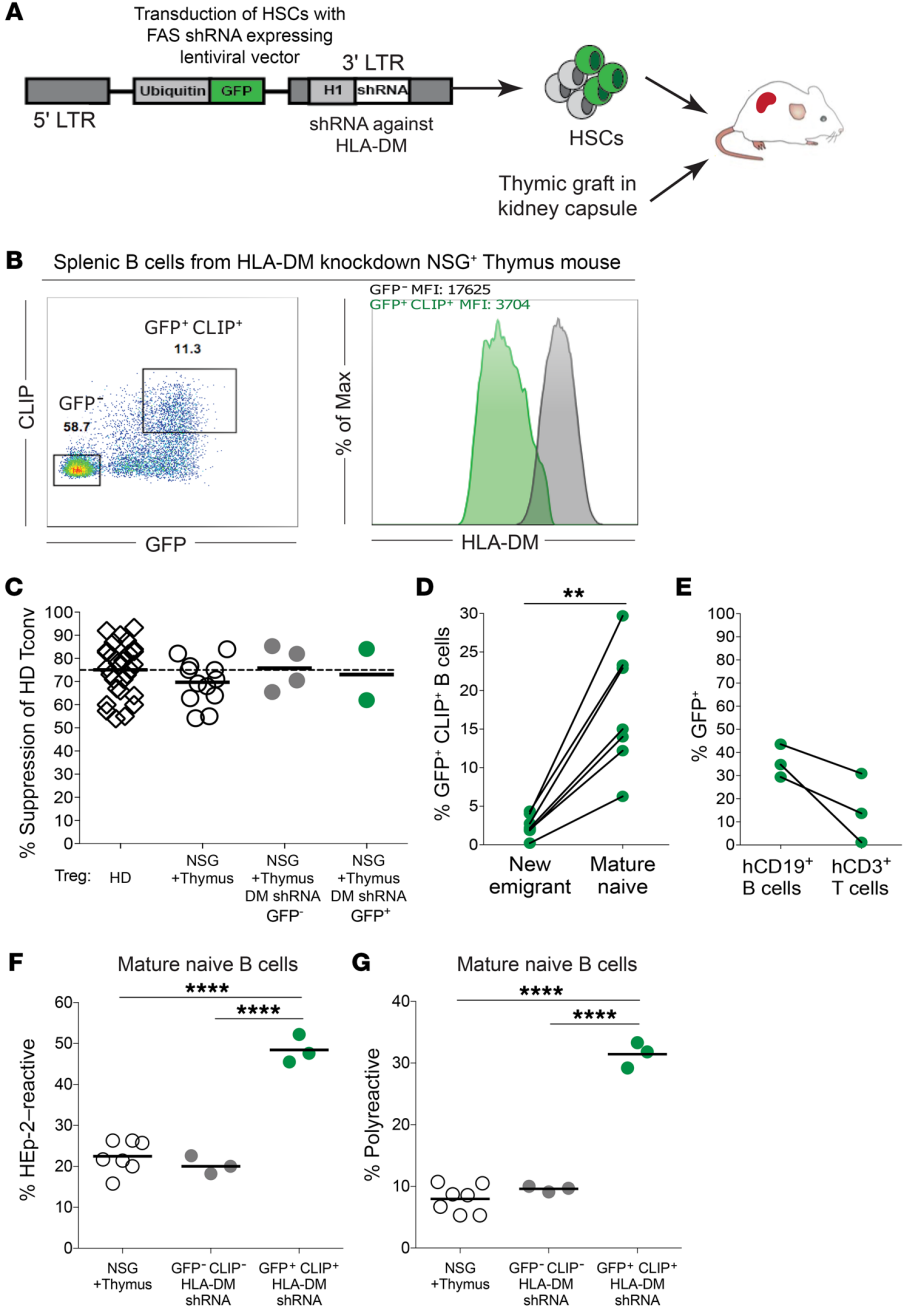
B cell–intrinsic antigen presentation is required for the establishment of the peripheral tolerance checkpoint. (**A**) CD34^+^ HSCs were transduced with a GFP-tagged lentivirus that allowed the expression of an shRNA against HLA-DM before engraftment into NSG + thymus humanized mice. LTR, long terminal repeat.(**B**) Representative flow cytometric analysis of CD19^+^ B cells isolated from the spleens of these modified NSG + thymus humanized mice. Left panel shows surface CLIP expression versus GFP expression; right panel shows intracellular staining for HLA-DM in sorted GFP^+^CLIP^+^ B cells versus GFP^–^ B cells. (**C**) Suppressive capacity of GFP^–^ and GFP^+^ Tregs from DM-knockdown NSG + thymus humanized mice compared counterpart Tregs from HDs, NSG humanized mice, and NSG + thymus humanized mice. Tregs from DM-knockdown humanized mice were suppressive regardless of GFP expression. (**D**) Proportions of GFP^+^CLIP^+^ B cells in the new emigrant/transitional and mature naive B cell compartments. (**E**) Proportions of GFP^+^ B cells and GFP^+^ T cells in the 3 HLA-DM–knockdown NSG + thymus humanized mice from which recombinant Abs were cloned from single B cells. The frequencies of (**F**) HEp-2–reactive and (**G**) polyreactive mature naive B cells from sorted GFP^+^CLIP^+^ B cells were determined and compared with those of GFP^–^ (unmodified) B cells from the same mice. Each symbol represents a mouse, and the average is shown with a bar. **P < 0.01 and ****P < 0.0001, by Kruskal-Wallis test.

While the impaired MHC class II expression on B cells from patients with BLS may interfere with the removal of autoreactive clones in the periphery, altered MHC class II expression in the thymus may also affect T cell selection and the TCR repertoires of T cells and Tregs involved in the regulation of this checkpoint. To further assess whether MHC class II–dependent self-antigen presentation by autoreactive B cells is critical for their removal, we designed a model in which human fetal HSCs were first transduced by a GFP-tagged lentivirus expressing an shRNA against *HLA-DM* prior to engraftment into NSG + thymus humanized mice (Figure 6A). HLA-DM is an MHC class II–like, but nonpolymorphic, peptide editor that facilitates the release of the class II–associated invariant chain peptide (CLIP) and allows the loading of antigenic peptides onto MHC class II molecules (42, 43). We found that HLA-DM knockdown in B cells that developed in NSG + thymus humanized mice led to the expression of CLIP–MHC class II rather than typical (self-) antigen peptide–MHC class II complexes on the surface of some GFP^+^ B cells, whereas CLIP expression was not detected on the surface of unmodified GFP^–^ B cells (Figure 6B). Intracellular staining of HLA-DM in GFP^+^CLIP^+^ B cells with a specific mAb revealed that HLA-DM expression was reduced by approximately 80% compared with expression in GFP^–^ B cells (Figure 6B). Overall, human immune reconstitution as well as total B and T cell proportions and numbers were not affected by HLA-DM knockdown in humanized mice (Supplemental Figure 8, A and B, and data not shown). MHC class II expression on developing thymocytes could also participate in the selection of CD4^+^ T cells (44). However, Tregs from HLA-DM–knockdown humanized mice appeared phenotypically and functionally similar to counterpart Tregs in regular NSG + thymus humanized mice, suggesting proper Treg development in this model (Figure 6C and Supplemental Figure 8C).

We found that B cells with silenced HLA-DM drastically increased in the periphery: GFP^+^CLIP^+^ cells accounted for less than 5% of the new emigrant/transitional B cell compartment but represented 10%–30% (average 17.6% ± 8.0%) of the mature naive B cell population (*P* = 0.001, Figure 6D). To assess the B cell tolerance status while avoiding a putative effect of T cell lineage alteration by HLA-DM inhibition, we specifically chose to test the reactivity of Abs expressed by GFP^–^ and GFP^+^CLIP^+^ B cells in 3 DM-knockdown NSG + thymus humanized mice, 2 of which had T cell compartments containing less than 30% GFP^+^ cells and 1 with virtually none (Figure 6E). The central B cell tolerance checkpoint was unaffected by HLA-DM inhibition because the GFP^+^CLIP^+^ new emigrant/transitional B cells from all 3 NSG + thymus humanized mice were not enriched for polyreactivity or antinuclear reactivity (Supplemental Figure 8, D and E). In contrast, we found that GFP^+^CLIP^+^ mature naive B cell populations contained significantly higher proportions of autoreactive and polyreactive clones, whereas unmodified GFP^–^ mature naive B cells from the same mice displayed low HEp-2 reactivity and polyreactivity, similar to B cells from regular NSG + thymus humanized mice (Figure 6, F and G, and Supplemental Figure 8, F and G). In addition, we observed that higher frequencies of antinuclear specificities accumulated in the GFP^+^CLIP^+^, but not the GFP^–^, mature naive B cell pools, implying that certain autoreactive clones specific for nuclear self-antigens that escaped central tolerance may have been continually suppressed in the periphery (Supplemental Figure 8, H and I). Moreover, reactivity differences between GFP^+^CLIP^+^ versus GFP^–^ B cells excluded a putative effect of MHC class II inhibition in T cell development, because this would have affected the peripheral selection of both GFP^+^ and GFP^–^ B cells, which is not what we observed. Of note, *HLA-DM* silencing occurred only in B cells but not T cells in 1 of the humanized mice and was sufficient to disrupt peripheral autoreactive B cell selection, thereby ruling out the involvement of putative defects in T cell development as a result of impaired MHC class II function in thymocytes (44). We conclude that the inhibition of MHC class II–mediated self-antigen presentation by autoreactive B cells resulted in a failure to repress these cells and their accumulation in the periphery.

### Human T cells that develop in an HLA-mismatched human thymic graft fail to suppress peripheral autoreactive B cell expansion.

**Figure 7:**
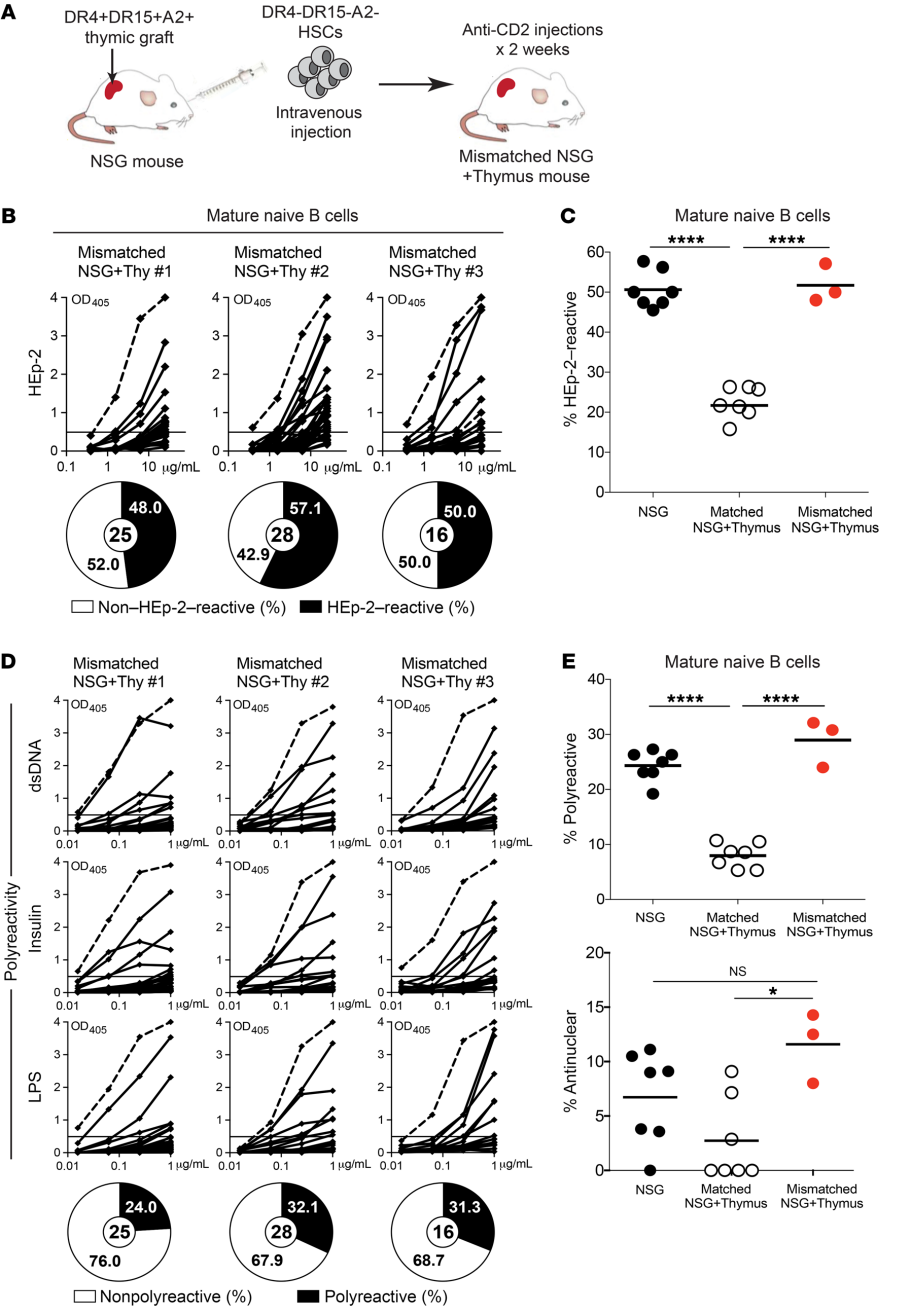
Peripheral B cell selection requires cognate interactions between B cells and T cells. (**A**) Thymic graft from 1 fetal donor was cotransplanted with HSCs from a different HLA-mismatched fetus into an NSG mouse. Two 50 μg doses of anti-CD2 were injected after the surgery to deplete initial thymocytes in the thymic graft. (**B**) HEp-2 reactivity of recombinant Abs cloned from single mature naive B cells from 3 HLA-mismatched NSG + thymus humanized mice were assessed by ELISA. (**C**) Summary of the frequencies of HEp-2–reactive mature naive B cells in mismatched NSG + thymus humanized mice compared with regular NSG + thymus and NSG humanized mice. Each symbol represents a mouse, and the average is shown with a bar. (**D**) Polyreactivity of recombinant Abs cloned from single mature naive B cells from 3 HLA-mismatched NSG + thymus (NSG+Thy) humanized mice was assessed by ELISA. (**E**) Summaries of the frequencies of polyreactive and antinuclear clones in mismatched NSG + thymus humanized mice compared with regular NSG + thymus and NSG humanized mice. *P < 0.01 and ****P < 0.0001, by Kruskal-Wallis test.

We established an additional NSG + thymus humanized mouse model in which the engrafted fetal HSCs and thymic graft came from MHC-discordant donors, so that T cells developed in a human thymic environment but were educated on human MHC molecules different from those expressed on B cells (Figure 7A). In the first set of these HLA-mismatched NSG + thymus humanized mice, we cotransplanted HSCs from an HLA-DR4^–^HLA-DR15^–^HLA-A2^–^ fetal donor and thymic fragments from an HLA-DR4^+^HLA-DR15^+^HLA-A2^+^ fetal donor (Figure 7A). To minimize chimerism, we treated these mice with anti–human CD2 Abs to deplete initial thymocytes and mature T cells from the thymic graft for 2 weeks following thymic implantation, as previously reported (45). The HLA-A2 genotype difference between the 2 fetal donors was useful to confirm the HSC-derived origin of peripheral T cells by flow cytometry using a commercially available Ab against HLA-A2; all peripheral T cells in mismatched NSG + thymus humanized mice had the same HLA-A2 phenotype as peripheral B cells, demonstrating that these human T cells were selected in an MHC-discordant thymic environment (Supplemental Figure 9A and ref. 46). Altogether, 3 combinations of HLA-mismatched NSG + thymus humanized mice were generated using tissues from 6 fetal donors (Supplemental Table 4). The human immune reconstitution, B and T cell proportions and numbers, and Treg phenotype and suppressive function were all similar between HLA-mismatched and regular HLA-matched NSG + thymus humanized mice (Supplemental Figure 9, B–F), and we observed no developmental abnormalities in T cells or Tregs.

Central B cell tolerance in these HLA-mismatched NSG + thymus humanized mice was normal (Supplemental Figure 9, G and H). However, the frequencies of autoreactive and polyreactive mature naive B cells expanded by approximately 2.5-fold in each of the 3 HLA-mismatched NSG + thymus humanized mice compared with counterpart cells in HLA-matched humanized mice (*P* < 0.05; Figure 7, B–E); antinuclear reactivities in the mature naive B cell pool of HLA-mismatched NSG + thymus humanized mice were also elevated in comparison with antinuclear reactivities in HLA-matched humanized mice (*P* < 0.05; Figure 7E). We therefore conclude that thymic T cell and Treg education needs to be established on the same MHC class II molecules as those expressed by peripheral B cells in order to inhibit autoreactive B cell accumulation in the periphery.

## Discussion

We report here that negative selection required thymus-derived Tregs and cognate recognition of MHC class II–restricted self-peptides presented by B cells, whereas positive selection acted directly on some naive B cells through BCR-dependent expansion of clones. Indeed, we showed the requirement of an MHC-matched thymus and Tregs for the successful prevention of autoreactive naive B cell accumulation in the periphery, whereas central B cell tolerance was functional in both NSG and NSG + thymus humanized mice and therefore did not depend on the presence of an autologous thymic graft. This latter observation is consistent with a prior study of patients with immunodeficiency, which demonstrated that the removal of developing autoreactive B cells in the bone marrow was unaffected by the absence of T cells (47), because central B cell tolerance is controlled by B cell–intrinsic BCR and TLR pathways (3–7, 14, 48, 49). In contrast, lack of T cells in patients with *CD3D* and *CD3E* gene mutations resulted in a specific disruption of the peripheral B cell tolerance checkpoint, thus suggesting that T cells play an important role in preventing the accumulation of autoreactive clones in the mature naive B cell compartment (47). In agreement with this observation, we recently reported that the peripheral B cell tolerance checkpoint is not yet operational in second-trimester human fetuses in which B cells produced by the fetal liver precede the emergence of peripheral T cells (23). This second selection step further shapes the naive B cell compartment in healthy individuals and may silence autoreactive clones that recognize self-antigens prevalently expressed in the periphery, as suggested by the dependence of this step on the autoimmune regulator–mediated (AIRE-mediated) expression of peripheral tissue antigens (47). It remains to be determined whether T cells interact with autoreactive new emigrant/transitional B cells that escape central B cell tolerance and prevent their entry into the mature naive B cell compartment, or if they suppress autoreactive mature naive B cells that differentiated from new emigrant/transitional B cells and thwart the accumulation of these autoreactive clones in the periphery.

Which T cell subset regulates the peripheral B cell tolerance checkpoint? The involvement of Tregs was suggested by the analysis of patients with immunodysregulation, polyendocrinopathy, enteropathy, X-linked (IPEX), who harbor dysfunctional Tregs due to loss-of-function *FOXP3* mutations and show elevated frequencies of circulating autoreactive mature naive B cells (50). Adoptive cell transfer studies in mice have also shown a role for Tregs in the negative regulation of autoantibody secretion (51, 52). In addition, specific defects in this peripheral B cell tolerance checkpoint have been associated with either decreased Treg frequencies or impaired Treg-suppressive function in patients with CD40 ligand (CD40L), adenosine deaminase (ADA), dedicator of cytokinesis 8 (DOCK8), Wiskott-Aldrich syndrome protein (WASP), and lipopolysaccharide-responsive and beige-like anchor protein (LRBA) deficiencies and further support an important role for Tregs in preventing the expansion of autoreactive B cells in the periphery (27, 28, 40, 48, 53). The depletion of Tregs by anti-CD25 in NSG + thymus humanized mice, which preserved most other CD4^+^ and CD8^+^ T cell subsets, specifically abrogated the silencing of autoreactive clones in the mature naive B cell compartment and therefore further indicates an important role for Tregs in controlling the peripheral B cell tolerance checkpoint. Of note, it is unclear if lymphopenia in humanized mouse models may alter B cell selection mechanisms normally occurring at steady state in an individual and whether it might further enhance the requirement of Tregs for the establishment of the peripheral B cell tolerance checkpoint. On the other hand, our observations may have a particular relevance for a variety of clinical settings in which B cell lymphopenia is an underlying condition such as autoimmune and autoinflammatory diseases.

A requirement of MHC class II expression for the establishment of a functional peripheral B cell tolerance checkpoint was first suggested by the study of a patient with classical BLS with *CIITA* gene mutations and a low CD4^+^ T cell count, who showed accumulation of self-reactive mature naive B cells (40). We now show that the absence of MHC class II expression on B cells of patients with atypical BLS or the inability of B cells to present (self)-antigen on MHC class II molecules induced by HLA-DM knockdown both interfered with the curtailing of autoreactive B cells in the periphery. The requirement of MHC class II–dependent self-antigen presentation to prevent the emergence of autoreactive naive B cells infers cognate interactions of these B cells with CD4^+^ T cells expressing autoreactive TCRs. In agreement with this hypothesis, human T cells and Tregs that developed in an HLA-mismatched thymic graft failed to ensure a functional peripheral B cell tolerance checkpoint, whereas these T cells and Tregs were educated in a human thymic environment that led to normal Treg-suppressive function and phenotype. These observations may explain why the transplantation of an HLA-unmatched thymus graft as a treatment for patients with complete DiGeorge syndrome, who lack a functional thymus and T cells, results in the production of T cells but also the appearance of post-transplantation autoimmune adverse events likely caused by the accumulation of autoreactive B cells in the periphery (54, 55). Since Tregs have been shown to express TCRs that are biased toward the recognition of self-antigens (56–59), they may regulate the peripheral B cell tolerance checkpoint through TCR–MHC class II interaction with autoreactive naive B cells in the periphery. Alternatively, Tregs may control peripheral B cell selection by repressing other autoreactive conventional T cells that escape central thymic tolerance and promote autoreactive B cell survival in the periphery. These 2 scenarios are not mutually exclusive and may both contribute to the establishment of peripheral B cell tolerance. Although the mechanisms by which the accumulation of autoreactive B cells is inhibited by Tregs in the periphery remain to be investigated in polyclonal model systems with human B and T cells, mouse studies suggest a Fas/Fas ligand–dependent death mechanism for B cells expressing a transgenic self-reactive BCR upon interaction with transgenic T cells expressing CD40L (12, 13).

Contrasting with negative autoreactive B cell selection in the periphery that requires the presence of an autologous human thymus, we found that the expansion of naive B cells occurred normally in NSG humanized mice and therefore did not rely on cognate interactions with T cells. While B cell positive selection may promote maintenance without the proliferation of B cells through maturation in the periphery, our assessment of positive selection was limited to clones that had undergone positive selection through proliferation in the mature naive B cell compartment. Since the BCR repertoire of expanded B cells is significantly different from that of nonexpanded clones, recognition of peripheral antigens likely plays an important role in this process, as previously suggested by transgenic mouse models (11). Although self-antigens positively select B-1 B cells in mice (60), they are not likely to promote peripheral B cell positive selection, because expanded clones in HDs and humanized mice represented up to 50% of the mature naive B cell compartment, a frequency much higher than that of autoreactive clones. In addition, positively selected mature naive B cells expressed BCRs with shorter CDR3s containing less positively charged amino acids, whereas self-reactive BCRs normally display long and positively charged CDR3s (1). The enrichment in VH3-encoded Abs in positively selected mature naive B cells has recently been associated with anti–commensal bacteria reactivity and suggests instead that microbiota antigens may contribute to clonal expansion in the mature naive B cell compartment (23, 61).

In conclusion, we report that the establishment of human peripheral B cell tolerance requires Tregs with suppressive function and antigen presentation by autoreactive naive B cells. While cognate self-antigen recognition appears to represent a crucial step in the proper purging of developing autoreactive clones from the circulating B cell repertoire, additional studies are warranted to investigate the precise cellular and molecular mechanisms that control this peripheral B cell tolerance checkpoint.

## Methods

### HDs and patients.

Blood samples were collected from HDs at the Yale School of Medicine. Patient BLS02 had a homozygous mutation on her *RFXAP* gene (exon 3 c.751 C>T, leading to a premature Gln251Stop codon). Patient BLS3 had a homozygous mutation on her *RFXANK* gene (intron 4 c.271+1delGinsTCAC, leading to a splice site shift).

### Fetal tissues.

Tissues from 105- to 135-day-old human fetuses, including bone marrow, spleen, liver, and thymus tissues, were obtained from the Laboratory of Developmental Biology at the University of Washington (Seattle, Washington, USA). Fetal samples with chromosomal abnormalities and those that tested positive for the *PTPN22* 1858T risk allele were excluded (5, 15).

### Humanized mice.

Immunodeficient NOD*-scid* common γ chain–deficient (NSG) mice were purchased from The Jackson Laboratory (stock no. 005557). Ten-week-old female NSG mice were used in thymic transplantation surgeries to generate NSG + thymus humanized mice, because human immune engraftment was more efficient in female hosts as previously reported (17). HSCs and thymic tissues from both male and female fetuses were used in the generation of NSG and NSG + thymus humanized mice. Human CD34^+^ cells were purified from fetal liver and bone marrow by density-gradient centrifugation followed by positive immunomagnetic selection with anti–human CD34 microbeads (Miltenyi Biotec). To generate NSG humanized mice, newborn pups (within the first 3 days of life) were sublethally irradiated (x-ray irradiation with the Precision X-Ray X-RAD 320 irradiator at 180 cGy), and 100,000–150,000 CD34^+^ HSCs resuspended in 20 μL PBS were injected into the liver with a 22 gauge needle (Hamilton Company). To generate NSG + thymus humanized mice, 9- to 11-week-old female NSG mice were conditioned with 2.5 Gy total body irradiation the day before surgery. Human fetal thymus fragments measuring approximately 1 mm^3^ were then implanted under the kidney capsule of the recipient mice. CD34^+^ fetal HSCs (200,000–300,000 cells) from the same donor were injected i.v. shortly after the surgery. For HLA-mismatched NSG + thymus humanized mice, 50 μg anti-CD2 mAb (LSBio) was injected i.p. over a 2-week period (1 dose/week) to eliminate preexisting thymocytes and mature T cells from the thymic graft (45). All mice were used for experiments 10–12 weeks after HSC transplantation.

### Cell isolation and cell sorting.

B cells were purified from the spleens of humanized mice by positive selection using CD19 microbeads (Miltenyi Biotec). Enriched B cells were stained with Abs against IgM, CD19, CD27, CD10, and CD21 (listed in the Key Resources table in the supplemental materials). CD19^+^CD21^–/lo^CD10^+^IgM^hi^CD27^–^ new emigrant/transitional and CD19^+^CD21^+^CD10^–^IgM^+^CD27^–^ mature naive B cells were batch-sorted using a BD FACSAria sorter or were sorted into 96-well PCR plates as single cells. CD4^+^ T cells were enriched from the non–B cell fraction using the EasySep Human CD4^+^T cell enrichment kit (STEMCELL Technologies) and stained with Abs against CD3, CD4, CD25, and CD127 (also listed in the Key Resources table in the supplemental materials). CD3^+^CD4^+^CD25^hi^CD127^lo^ Tregs and CD3^+^CD4^+^CD25^–/lo^ CD127^+^ Tconv cells were then batch-sorted for in vitro suppression assays and/or immunosequencing.

### cDNA, reverse transcription PCR, Ab production, and purification.

RNA from single B cells was reverse-transcribed in the original 96-well plate in 12.5 μL reactions containing 100 U Superscript II RT (Gibco BRL, Thermo Fisher Scientific) for 45 minutes at 42°C. Reverse transcription PCR (RT-PCR) reactions, primer sequences, cloning strategy, expression vectors, Ab expression, and purification were carried out as previously described (1).

### KREC assay.

The ratio of KREC joints (signal joint) to the Jκ-Cκ recombination genomic joints (coding joint) was determined as previously described (25).

### ELISAs and indirect immunofluorescence staining.

Ab concentrations and reactivity were measured as described before (1). Abs were considered polyreactive when they recognized all 3 analyzed antigens: dsDNA, insulin, and LPS. The cutoff OD_405_ corresponded to twice the average OD_405_ ELISA value of all tested clones and was approximatively 0.5. For indirect immunofluorescence assays, HEp-2 cell–coated slides (Bion Enterprises) were incubated in a moist chamber at room temperature with purified recombinant Abs at 50–100 μg/mL and detected with FITC-conjugated goat anti–human IgG. Mouse and human serum BAFF concentrations were determined by ELISA according to the manufacturer’s instruction (R&D Systems).

### B cell and Treg immunophenotyping.

After CD19 microbead magnetic separation, non–B cell fractions from HDs or humanized mice were stained with Abs against the Treg markers CD3, CD4, CD25, and CD127 as well as the surface markers of interest CXCR3, CCR5 ICOS, HLA-DR, CCR7, and CTLA-4. Abs against intracellular markers of interest including FOXP3, TCF1, LEF1, BCL2, and Ki67 were added after fixation and permeabilization of T cells (Invitrogen, Thermo Fisher Scientific). For the phenotyping of B cells derived from the HLA-DM–knockdown NSG + thymus humanized mice, magnetically enriched B cells were stained with anti-surface CLIP Ab, fixed and permeabilized, and then stained with anti–HLA-DM Ab. All mAbs used are listed in the Key Resources table in the supplemental materials. Flow cytometry was performed on a BD LSR II flow cytometer, and the data were analyzed with FlowJo software.

### Treg suppression assays.

CD3^+^CD4^+^CD25^–/lo^CD127^+^ Tconv cells were labeled with CellTrace CFSE (Invitrogen, Thermo Fisher Scientific). Cocultures of Tregs and Tconv cells at a 1:1 ratio were stimulated with the Treg Suppression Inspector Human kit (Miltenyi Biotec), which contains anti-CD2/anti-CD3/anti-CD28, at a 1 bead/1 cell ratio. The proliferation of viable Tconv cells was analyzed by CFSE dilution using flow cytometry 3.5 days after stimulation.

### Confocal microscopy.

Frozen spleens from NSG and NSG + thymus humanized mice were serially sectioned (3 μm). Tissue was fixed in 4% paraformaldehyde, permeabilized with 0.2% Triton X-100, and then blocked with 10% normal donkey serum. The tissue was then stained with primary Abs against FOXP3 (Invitrogen, Thermo Fisher Scientific, 14-476-82), CD38 (Abcam, ab108403), and CD21 (Abcam, ab237981), followed by fluorochrome-conjugated secondary Abs specific for the primary species and isotypes (Invitrogen, Thermo Fisher Scientific). Directly conjugated CD4 (Abcam, ab19647) and CD20 (eBioscience, 53-0202-82) Abs were then added. Last, DAPI (Invitrogen, Thermo Fisher Scientific) was applied for nuclear staining. Images were acquired at 12 bit depth and 1024 × 1024 pixel size using an SP8 laser scanning confocal microscope (Leica). The number of B (CD20^+^) cells next to Tregs (CD4^+^FOXP3^+^ cells) was manually counted in each field of view.

### Lentivirus and HSC transduction.

The preparation of lentiviral vectors carrying shRNA and transduction of HSCs with lentiviruses were performed as previously described (14, 15). Briefly, a DNA fragment containing the H1 promoter and an shRNA sequence against HLA-DM (5′-GATCCCCGCATTGTTCTCATCATCTATTCAAGAGATAGATGATGAGA ACAATGCTTTTTA-3′) was generated by double digestion of the pSUPER plasmid and then subcloned within the 3′ long terminal repeat of the pTRIP-Ubi-GFP vector. Lentiviral particles were produced by transient transfection of 293T cells and concentrated approximately 100-fold by ultracentrifugation. Fetal HSCs were transduced with lentivirus expressing the HLA-DM shRNA and cultured overnight in Stemline Medium (MilliporeSigma) supplemented with recombinant human SCF, FLT3-L, and IL-3 (all from R&D Systems) prior to engraftment into NSG mice.

### BCR repertoire analysis.

Ig sequences from single B cells were analyzed by Ig BLAST comparison with GenBank using the NCBI’s IgBLAST server (http://www.ncbi.nlm.nih.gov/igblast/). Heavy-chain complementarity determining region 3 was defined as the interval between amino acids at position 94 in the VH framework 3 and the conserved tryptophan at position 103 in J_H_ segments. The sequences are included in Supplemental Table 1. For batch-sorted mature naive B cells, dry pellets frozen at –80°C were sent for IgH immune sequencing at Adaptive Biotechnologies (Seattle, Washington, USA).

### RNA-Seq analysis.

Frozen dry pellets of 50,000–100,000 sorted CD3^+^CD4^+^CD25^hi^CD127^–/lo^ Tregs from HDs and humanized mice were sent to Q2 Solutions/EA Genomics for isolation and sequencing of RNA using their TruSeq Stranded mRNA protocol. Read counts were analyzed by EA Genomics’ BI300 bioinformatics package. Differential expression of genes was analyzed in RStudio using the DEseq2 package (Bioconductor). An absolute fold change of 2 and an adjusted *P* value corresponding to an FDR of 0.01 were set as thresholds for significant differential expression. Volcano plots were graphed using the ggplot package in RStudio. Gene ontogeny/pathway differences were analyzed using the functional annotation tool of the DAVID (Database for Annotation, Visualization, and Integrated Discovery) bioinformatics database (david-d.ncifcrf.gov).

### Data availability.

ImmunoSEQ data and RNA-Seq data are available on Adaptive Biotechnologies’ immuneACCESS website (https://clients.adaptivebiotech.com/pub/chen-2021-jci; DOI: 10.21417/JWC2021JCI) and in the NCBI’s Gene Expression Omnibus (GEO) database (GEO GSE186881), respectively. The sequencing data, including 146,604 IgH sequences from 4 HDs, 133,800 IgH sequences from 6 NSG humanized mice, and 45,223 IgH sequences from 5 NSG + thymus humanized mice, are publicly available on Adaptive Technologies’ immuneACCESS website (https://clients.adaptive.biotech.com; login credentials to see the data, email: chen-review@adaptivebiotech.com; password: chen2021review).

### Statistics.

Statistical analysis was performed using GraphPad Prism, version 7.03 (GraphPad Software). Unless otherwise noted, differences in B cell reactivities were analyzed by Kruskal-Wallis tests that were more appropriate than multiple, parallel Mann-Whitney *U* tests. Comparisons involving HSC donor–matched pairs reported in Figure 6, C and D, were analyzed by paired Student’s *t* test. Other multiple-group comparisons were corrected for statistical significance with the Bonferroni-Dunn method. Data are presented as mean ± SEM unless otherwise indicated in the figure legends. A *P* value of 0.05 or less was considered significant.

### Study approval.

Blood samples were collected from HDs with written consent in accordance with the Yale School of Medicine IRB protocol number 0906005336. Blood samples from patient BLS02 and patient BLS03, who provided written consent, were collected and shipped by Catharina Schuetz (Department of Pediatrics and Adolescent Medicine, Ulm University Medical Center, Ulm, Germany).

## Author contributions

EM, JWC, and JNS conceptualized the study and designed the methodology. JWC, JNS, NT, JS, FA, DB, DP, RG, JMB, FRD, MV, CS, EMJ, CP, ASS, KS, MRC, and LM performed studies. JWC and EM wrote the original draft of the manuscript. CS and ASS provided resources. FEM acquired funding. EM supervised the work.

## Supplementary Material

Supplemental data

Supplemental Table 1

## Version 1 11/23/2021

In-Press Preview

## Version 2 01/18/2022

Electronic publication
