# Supplementary material for: Positive and negative selection shape the human naive B cell repertoire
Source: J Clin Invest. 2022 Jan 18;132(2):e150985. doi: 10.1172/JCI150985 (PMC8759783; doi:10.1172/JCI150985)
Supplement: Supplemental data [file jci-132-150985-s143.pdf]

Supplementary Materials for

Positive and negative selection shape the human naïve B cell repertoire

Jeff W. Chen, Jean-Nicolas Schickel, Nikolaos Tsakiris, Joel Sng, Florent Arbogast, Delphine Bouis, Daniele Parisi, Ruchi Gera, Joshua M. Boeckers, Fabien R. Delmotte, Margaret Veselits, Catharina Schuetz, Eva-Maria Jacobsen, Carsten Posovszky, Ansgar S. Schulz, Klaus Schwarz, Marcus R. Clark, Laurence Menard, and Eric Meffre

Correspondence to: eric.meffre@yale.edu

This PDF file includes:

Figs. S1 to S9
Key resources

Other Supplementary Materials for this manuscript include the following:

Table S1 (Excel file). Repertoire and reactivity of antibodies cloned from new emigrant/transitional and mature naïve B cells from humanized mice and patients
Table S2. List of TCR Vβ genes that were differentially expressed in batch-sorted Tregs from NSG vs. NSG+Thymus humanized mice
Table S3. Summary of BLS patient characteristics
Table S4. HLA Class II and Class I alleles of HSC and thymic graft donors used to generate three sets of HLA-mismatched NSG+Thymus humanized mice

\

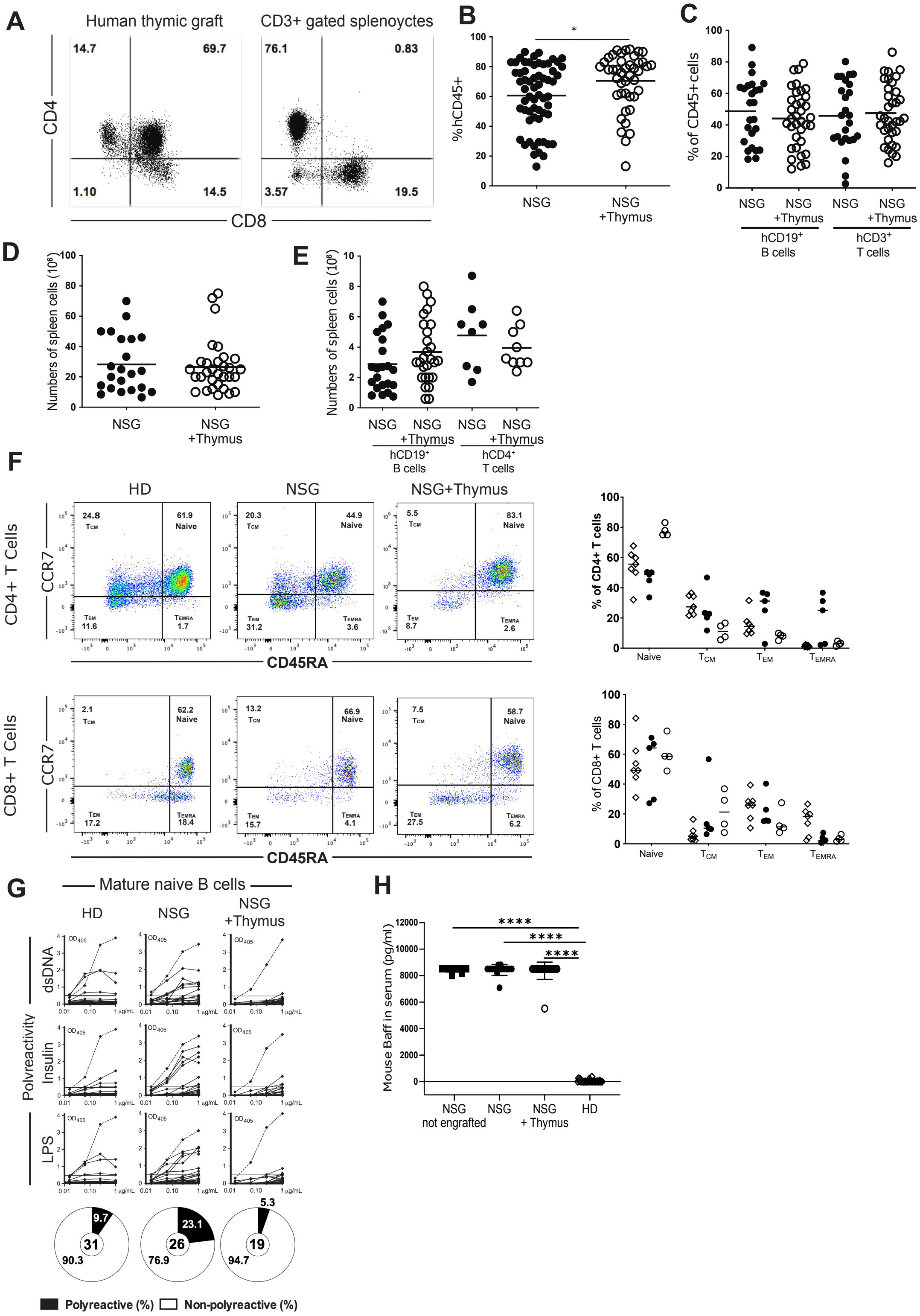
A
Human thymic graft
CD3+ gated splenoyctes
CD4
CD8
14.7
69.7
1.10
14.5
76.1
0.83
3.57
19.5
B
% hCD45+
NSG
NSG +Thymus
C
% of CD45+cells
NSG
NSG +Thymus
hCD19+ B cells
hCD3+ T cells
D
Numbers of spleen cells (10^6)
NSG
NSG +Thymus
E
Numbers of spleen cells (10^6)
NSG
NSG +Thymus
hCD19+ B cells
hCD4+ T cells
F
HD
NSG
NSG+Thymus
CD4+ T Cells
CCR7
CD45RA
24.8 TCM
61.9 Naive
TEM 11.6
TEMRA 1.7
20.3 TCM
44.9 Naive
TEM 31.2
TEMRA 3.6
5.5 TCM
83.1 Naive
TEM 8.7
TEMRA 2.6
% of CD4+ T cells
Naive
TCM
TEM
TEMRA
CD8+ T Cells
2.1 TCM
62.2 Naive
TEM 17.2
TEMRA 18.4
13.2 TCM
66.9 Naive
TEM 15.7
TEMRA 4.1
7.5 TCM
58.7 Naive
TEM 27.5
TEMRA 6.2
% of CD8+ T cells
G
Mature naive B cells
HD
NSG
NSG +Thymus
Polyreactivity
dsDNA
Insulin
LPS
OD405
1 µg/mL
9.7
31
90.3
23.1
26
76.9
5.3
19
94.7
Polyreactive (%)
Non-polyreactive (%)
H
Mouse Baff in serum (pg/ml)
****
NSG not engrafted
NSG
NSG + Thymus
HD

Figure S1. Immuno-phenotyping of NSG and NSG+Thymus humanized mice. (**A**) Representative flow cytometry analysis of engrafted human thymus (left) vs. splenocytes (right) shows developing $CD4^+CD8^+$ thymocytes in the thymic graft but not in the periphery. (**B**) Summary of human $CD45^+$ leukocyte engraftment in the blood of NSG (filled circles) and NSG+Thymus (open circles) humanized mice. (**C**) Summary of human $CD19^+$ B cells and $CD3^+$ T cells as % of $hCD45^+$ cells in the blood of NSG and NSG+Thymus humanized mice. Numbers of total splenocytes, CD19+ B cells and $CD4^+$ T cells in NSG (filled circles) and NSG+Thymus (open circles) humanized mice are displayed in (**D**) and (**E**), respectively. (**F**) Representative dot plots show the expression of CCR7 and CD45RA on gated $CD3^+CD4^+$ T cells (top) and $CD3^+CD8^+$ T cells (bottom) from a healthy donor (HD), and in NSG and NSG+Thymus humanized mice. The frequencies of naive T cells ($CD45RA^+CCR7^+$), central memory T cells (T_{CM}, $CD45RA^-CCR7^+$), effector memory T cells (T_{EM}, $CD45RA^-CCR7^-$) and $CD45RA^+$ effector memory cells (T_{EMRA}, $CD45RA^+CCR7^-$) for both $CD4^+$ and $CD8^+$ T cells from HD (open diamonds), NSG (filled circles) and NSG+Thymus (filled circles) humanized mice are represented on the right. (**G**) Recombinant antibodies cloned from mature naïve B cells from healthy donors (HD, n=13), NSG (n=7) and NSG+Thymus (n=7) humanized mice were tested by ELISA for polyreactivity. Dotted lines show the ED38 positive control. Horizontal lines show the cut-off OD_{405} for positive reactivity. For each individual or humanized mouse, the frequency of non-reactive (open area) and reactive (filled area) clones is summarized in a pie chart below, with the total number of clones tested indicated in the centers. (**H**) mouse BAFF concentrations were measured by ELISA in the sera of non-engrafted NSG mice, NSG and NSG+Thymus humanized mice and healthy donors (HD). ***$P < 0.001$ (Kruskal-Wallis test).

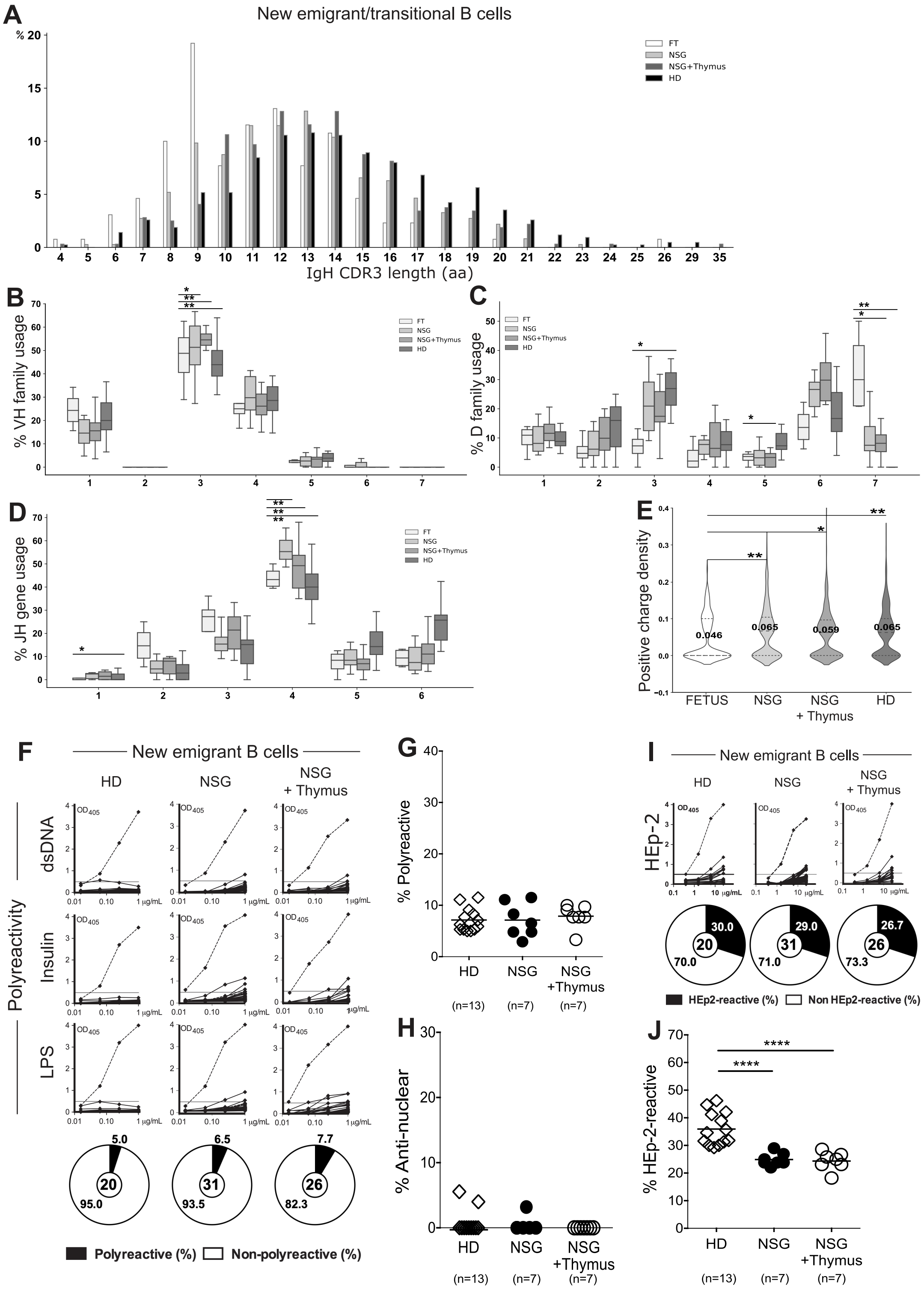
A
New emigrant/transitional B cells
FT
NSG
NSG+Thymus
HD
IgH CDR3 length (aa)
B
% VH family usage
C
% D family usage
D
% JH gene usage
E
Positive charge density
0.046
0.065
0.059
0.065
FETUS
NSG
NSG + Thymus
HD
F
New emigrant B cells
HD
NSG
NSG + Thymus
Polyreactivity
dsDNA
Insulin
LPS
OD_{405}
μg/mL
5.0
20
95.0
6.5
31
93.5
7.7
26
82.3
Polyreactive (%)
Non-polyreactive (%)
G
% Polyreactive
HD
NSG
NSG +Thymus
(n=13)
(n=7)
(n=7)
H
% Anti-nuclear
HD
NSG
NSG +Thymus
(n=13)
(n=7)
(n=7)
I
New emigrant B cells
HD
NSG
NSG + Thymus
HEp-2
30.0
20
70.0
29.0
31
71.0
26.7
26
73.3
HEp2-reactive (%)
Non HEp2-reactive (%)
J
% HEp-2-reactive
HD
NSG
NSG +Thymus
(n=13)
(n=7)
(n=7)

Figure S2. Functional central B cell tolerance in NSG and NSG+Thymus humanized mice. (**A**) IgH CDR3 length in amino acids, (**B**) VH family usage, (**C**) D family usage, (**D**) JH gene usage**,** (**E**) positive charge density (positive charges/CDR3 length) of IgH CDR3s from antibodies expressed by single new emigrant/transitional B cells isolated from 4 fetuses, 7 NSG and 7 NSG+Thymus humanized mice and 13 healthy donors (HD). *P < 0.05, **P < 0.01 (unpaired Z-tests). (**F**) Polyreactivity of recombinant antibodies cloned from single new emigrant/transitional B cells from NSG and NSG+Thymus humanized mice were assessed by ELISAs and compared with a representative healthy donor (HD). Dotted lines show the ED38 positive control. Horizontal lines show the cut-off OD_{405} for positive reactivity. For each subject, the frequency of non-reactive (open area) and reactive (filled area) clones is summarized in pie charts, with the total number of clones tested indicated in the centers. (**G**) Polyreactivity summary of new emigrant/transitional B cells of the indicated group. Each open diamond represents a healthy individual and each circle represents a humanized mouse. (**H**) Anti-nuclear reactivity in new emigrant/transitional B cells of the indicated group. Anti-nuclear reactivity was low in both humanized mouse models and reflects a normal central B cell tolerance. (**I**) HEp-2 reactivity of recombinant antibodies cloned from single new emigrant/transitional B cells from NSG and NSG+Thymus humanized mice were assessed by ELISAs and compared with a representative healthy donor. (**J**) Summary of HEp-2 reactivity in new emigrant/transitional B cells of the indicated group. ****P < 0.0001 (Kruskal-Wallis test).

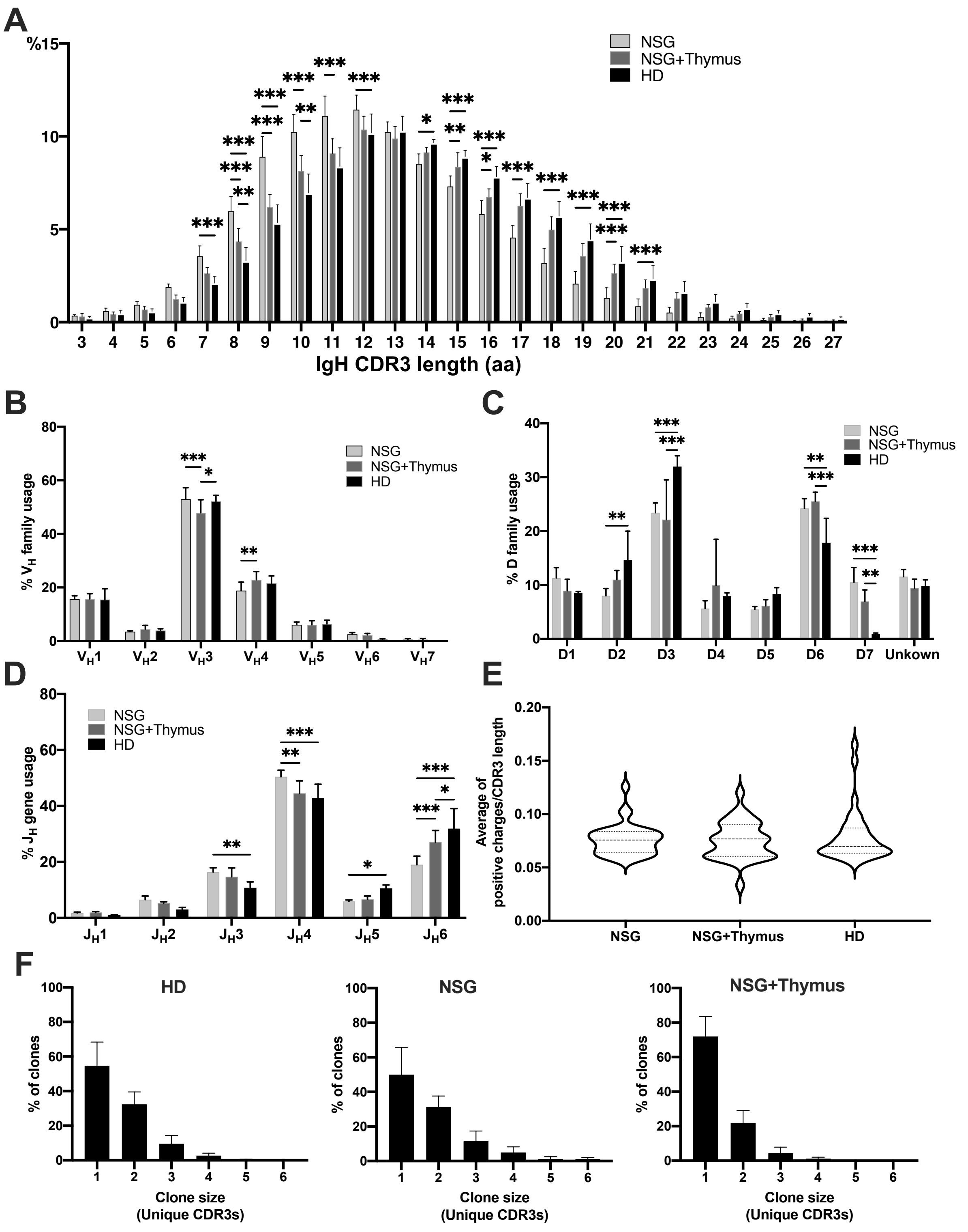
A
NSG
NSG+Thymus
HD
%15
10
5
0
3 4 5 6 7 8 9 10 11 12 13 14 15 16 17 18 19 20 21 22 23 24 25 26 27
IgH CDR3 length (aa)
B
% V_H family usage
V_H1 V_H2 V_H3 V_H4 V_H5 V_H6 V_H7
C
% D family usage
D1 D2 D3 D4 D5 D6 D7 Unkown
D
% J_H gene usage
J_H1 J_H2 J_H3 J_H4 J_H5 J_H6
E
Average of positive charges/CDR3 length
NSG NSG+Thymus HD
F
HD
NSG
NSG+Thymus
% of clones
Clone size (Unique CDR3s)

Figure S3. Characteristics of antibodies expressed by mature naïve B cells from NSG and NSG+Thymus humanized mice compared to healthy donors. (**A**) IgH CDR3 length in amino acids, (**B**) VH family usage, (**C**) D family usage, (**D**) JH gene usage**,** (**E**) average of positive charges/CDR3 length of antibodies expressed by mature naive B cells isolated from 6 NSG and 5 NSG+Thymus humanized mice and 4 healthy donors (HD). Analysis was performed with heavy chain sequences of mature naïve B cells (133,800 from NSG, 45,223 from NSG+Thymus and 146,604 from HD) obtained from Adaptive immunosequencing. (**F**) Identification of expanded clones in mature naïve B cells. Clone (unique CDR3s) size frequencies are represented for 6 NSG and 5 NSG+Thymus humanized mice and 4 HD. **P* < 0.05, ***P* < 0.01, ****P* < 0.001 (Two-way ANOVA followed by Tukey post hoc multi-comparison tests).

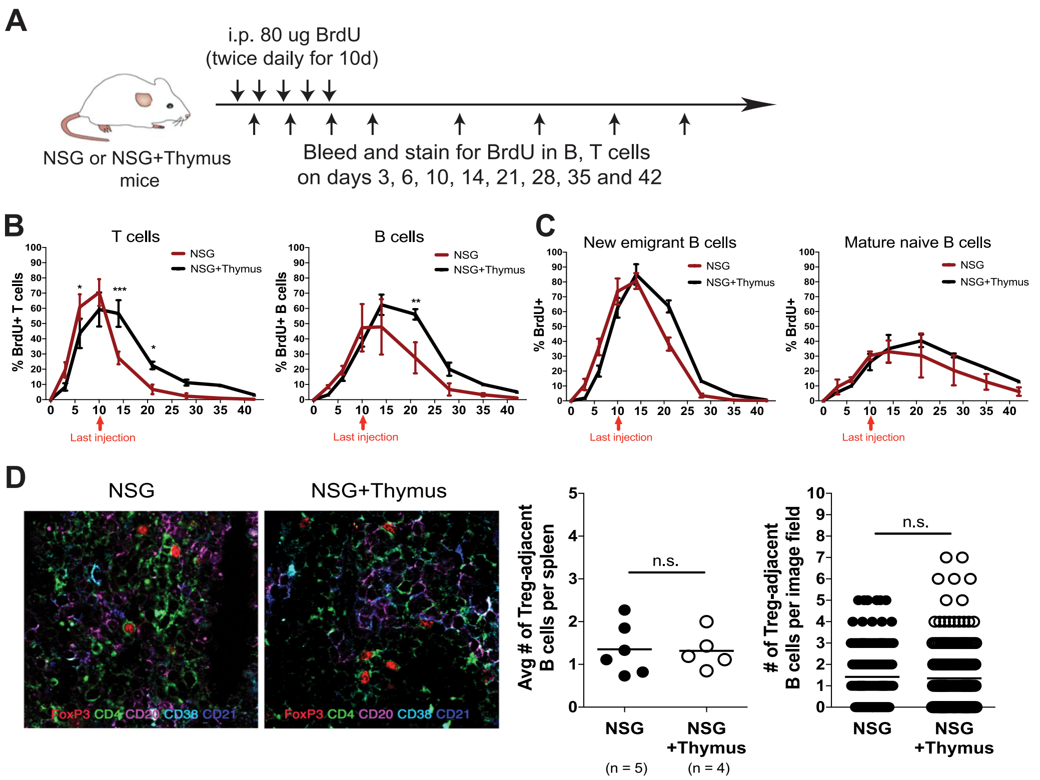

Figure S4. Evaluation of B and T cell turn over and spleen positioning in NSG and NSG+Thymus humanized mice. (**A**) Schematic diagram depicting the BrdU injection and staining. NSG and NSG+Thymus humanized mice were injected (i.p.) with 80 μg of BrdU twice a day for 10 days. Blood was collected and stained for immune cell markers and BrdU incorporation at the indicated time points. (**B**) Proportions of circulating BrdU+ T cells (left) and B cells (right) in NSG and NSG+Thymus humanized mice are plotted over time. Mean and standard deviation are indicated for each time point. *P < 0.05, **P < 0.01, ***P < 0.001 (ANOVA with Sidak's correction for multiple comparisons). (**C**) Proportions of circulating BrdU+ new emigrant/transitional (left) and mature naïve B cells (right). Mature naïve B cells displayed slower incorporation of BrdU and slower decay compared to new emigrant/transitional B cells. (**D**) Representative images (left) and summaries (right) of Treg and B cell association in the spleens of NSG and NSG+Thymus humanized as determined by confocal microscopy.

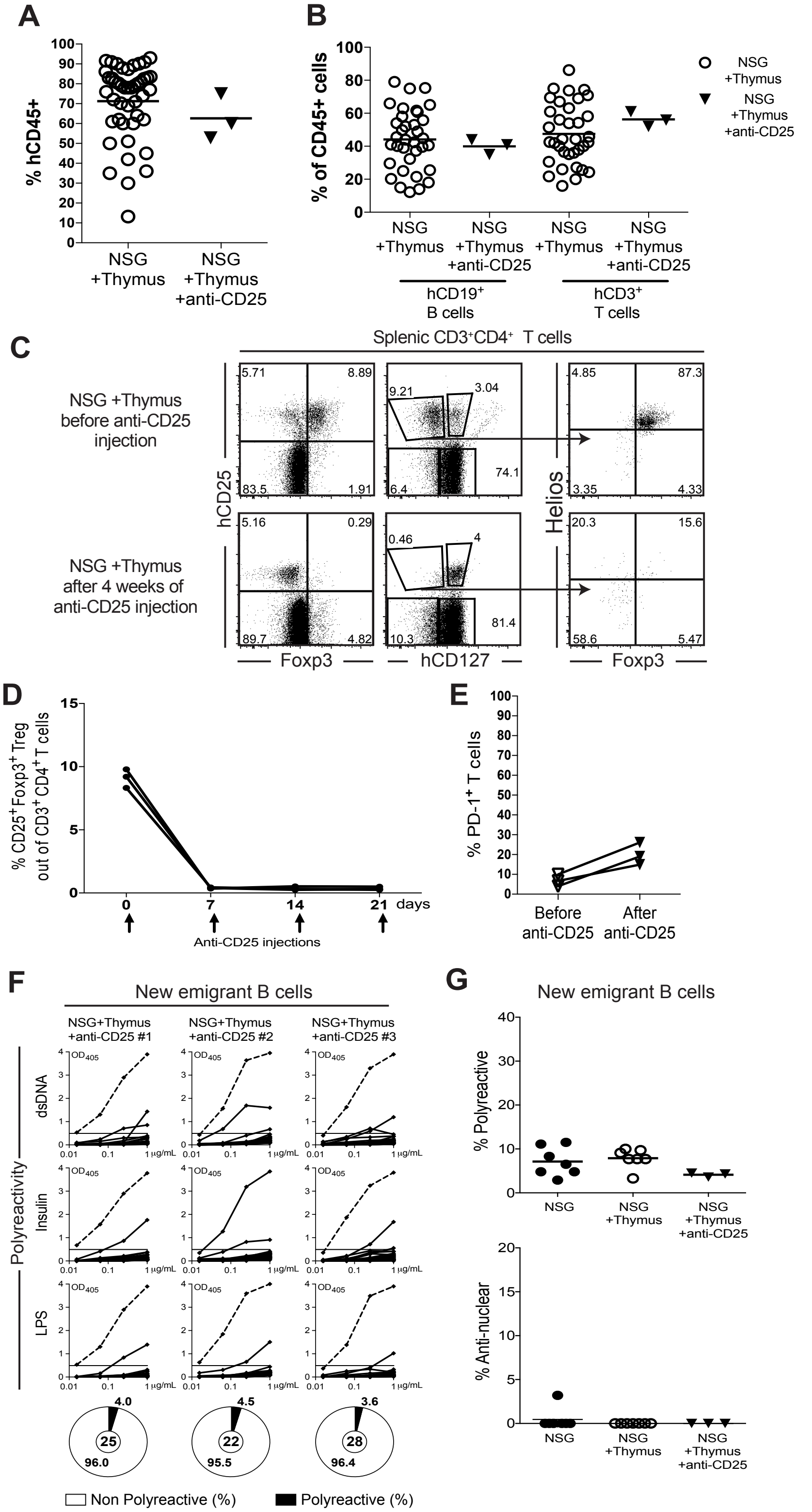
A
% hCD45+
NSG +Thymus
NSG +Thymus +anti-CD25
B
% of CD45+ cells
NSG +Thymus
NSG +Thymus +anti-CD25
hCD19+ B cells
hCD3+ T cells
C
Splenic CD3+CD4+ T cells
NSG +Thymus before anti-CD25 injection
NSG +Thymus after 4 weeks of anti-CD25 injection
hCD25
Helios
Foxp3
hCD127
5.71
8.89
83.5
1.91
9.21
3.04
6.4
74.1
4.85
87.3
3.35
4.33
5.16
0.29
89.7
4.82
0.46
4
10.3
81.4
20.3
15.6
58.6
5.47
D
% CD25+Foxp3+ Treg out of CD3+CD4+ T cells
days
Anti-CD25 injections
E
% PD-1+ T cells
Before anti-CD25
After anti-CD25
F
New emigrant B cells
NSG+Thymus +anti-CD25 #1
NSG+Thymus +anti-CD25 #2
NSG+Thymus +anti-CD25 #3
Polyreactivity
dsDNA
Insulin
LPS
OD405
μg/mL
4.0
25
96.0
4.5
22
95.5
3.6
28
96.4
Non Polyreactive (%)
Polyreactive (%)
G
New emigrant B cells
% Polyreactive
% Anti-nuclear
NSG
NSG +Thymus
NSG +Thymus +anti-CD25

Figure S5. NSG+Thymus humanized mice injected with mouse anti-human CD25 antibodies display functional central B cell tolerance. (**A**) Summary of human $CD45^+$ leukocyte engraftment in the blood of NSG+Thymus humanized mice injected or not with anti-CD25 antibodies. (**B**) Summary of human $CD19^+$ B cells and $CD3^+$ T cells as % of $hCD45^+$ cells in the blood of NSG+Thymus humanized mice injected or not with anti-CD25 antibodies. (**C**) Representative flow cytometry analysis of $CD25^{hi}Foxp3^+$ (left) or $CD25^{hi}CD127^{-/lo}$ (middle) Tregs gated on $CD3^+CD4^+$ T cells before and after the anti-CD25 regimen. HELIOS and FoxP3 expression (right) on gated $CD3^+CD4^+CD25^{hi}CD127^{-/lo}$ cells are also shown. (**D**) A time course assessment of the proportion of circulating $CD3^+CD4^+CD25^{hi}CD127^{-/lo}$ Tregs in NSG+Thymus humanized mice injected with anti-CD25 antibodies. Tregs are depleted within one week after the start of the regimen. (**E**) Proportion of PD-1^+ cells (gated on $CD3^+$ T cells) at the start and end of the anti-CD25 regimen. A modest increase in the frequency of PD-1^+ T cells was observed. (**F**) Polyreactivity of recombinant antibodies cloned from single new emigrant/transitional B cells from anti-CD25-injected NSG+Thymus humanized mice were assessed by ELISAs. Dotted lines show ED38-positive control. Horizontal lines show cut-off OD_{405} for positive reactivity. For each subject, the frequency of non-reactive (open area) and reactive (filled area) clones is summarized in pie charts, with the total number of clones tested indicated in the centers. (**G**) Summary of polyreactivity and anti-nuclear reactivity in the new emigrant/transitional B cell compartment of the indicated group. Each symbol represents an individual humanized mouse.

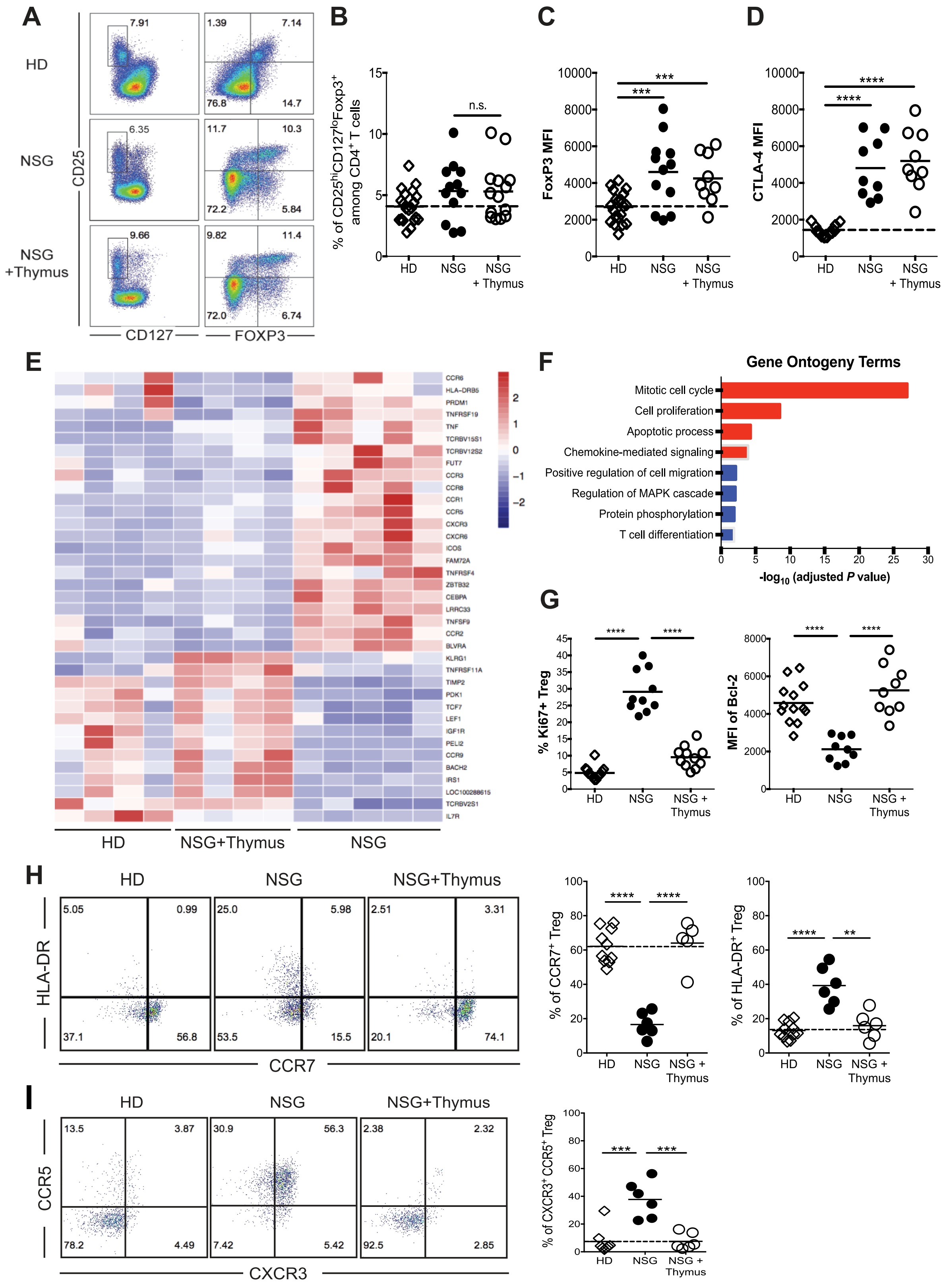

A
HD
NSG
NSG +Thymus
CD25
CD127
FOXP3
B
% of CD25hiCD127loFoxp3+ among CD4+ T cells
n.s.
C
FoxP3 MFI
D
CTLA-4 MFI
HD
NSG
NSG + Thymus
E
HD
NSG+Thymus
NSG
F
Gene Ontogeny Terms
Mitotic cell cycle
Cell proliferation
Apoptotic process
Chemokine-mediated signaling
Positive regulation of cell migration
Regulation of MAPK cascade
Protein phosphorylation
T cell differentiation
-log10 (adjusted P value)
G
% Ki67+ Treg
MFI of Bcl-2
H
HLA-DR
CCR7
% of CCR7+ Treg
% of HLA-DR+ Treg
I
CCR5
CXCR3
% of CXCR3+ CCR5+ Treg

Figure S6. Tregs from NSG and NSG+Thymus humanized mice are transcriptionally distinct. (**A**) Representative flow cytometry analysis of CD25 vs. CD127 and CD25 vs. FOXP3 in $CD4^+$ T cells from the blood of a healthy donor (HD), NSG and NSG+Thymus humanized mice. (**B**) Summary of the frequency of circulating $CD25^{hi}$ $CD127^{-/lo}$ $FOXP3^+$ Tregs (as % of $CD4^+$ T cells) in the indicated group. Summary of the MFI of (**C**) FOXP3 and (**D**) CTLA-4 expression in Tregs of the indicated group. ****P* < 0.001, *****P* < 0.0001, n.s. not significant (Student's t test). (**E**) Heatmap of a select group of genes differentially expressed by Tregs from NSG and NSG+Thymus humanized mice. In nearly all cases, expression of these genes in Tregs of healthy donors (HD) was similar to NSG+Thymus but not NSG humanized mice. (**F**) List of gene ontogeny (GO) terms differentially expressed in Tregs from NSG vs. NSG+Thus Tregs, ordered from the highest to lowest significance. GO terms that were more highly expressed in NSG Tregs are represented in red and those more highly expressed in NSG+Thymus Tregs in blue. (**G**) Summary of flow cytometry analysis of proliferation marker Ki67 and anti-apoptotic regulator Bcl-2 expression in Tregs from HD, NSG and NSG+Thymus humanized mice. *****P* < 0.0001 (Student's t test). Representative flow cytometry plots (left) and summary dot plots (right) of (**H**) CCR7 and HLA-DR or (**I**) CXCR3 and CCR5, expression in Tregs from HD, NSG, and NSG+Thymus humanized mice. ** *P* < 0.01; *****P* < 0.001, *****P* < 0.0001 (Kruskal-Wallis test).

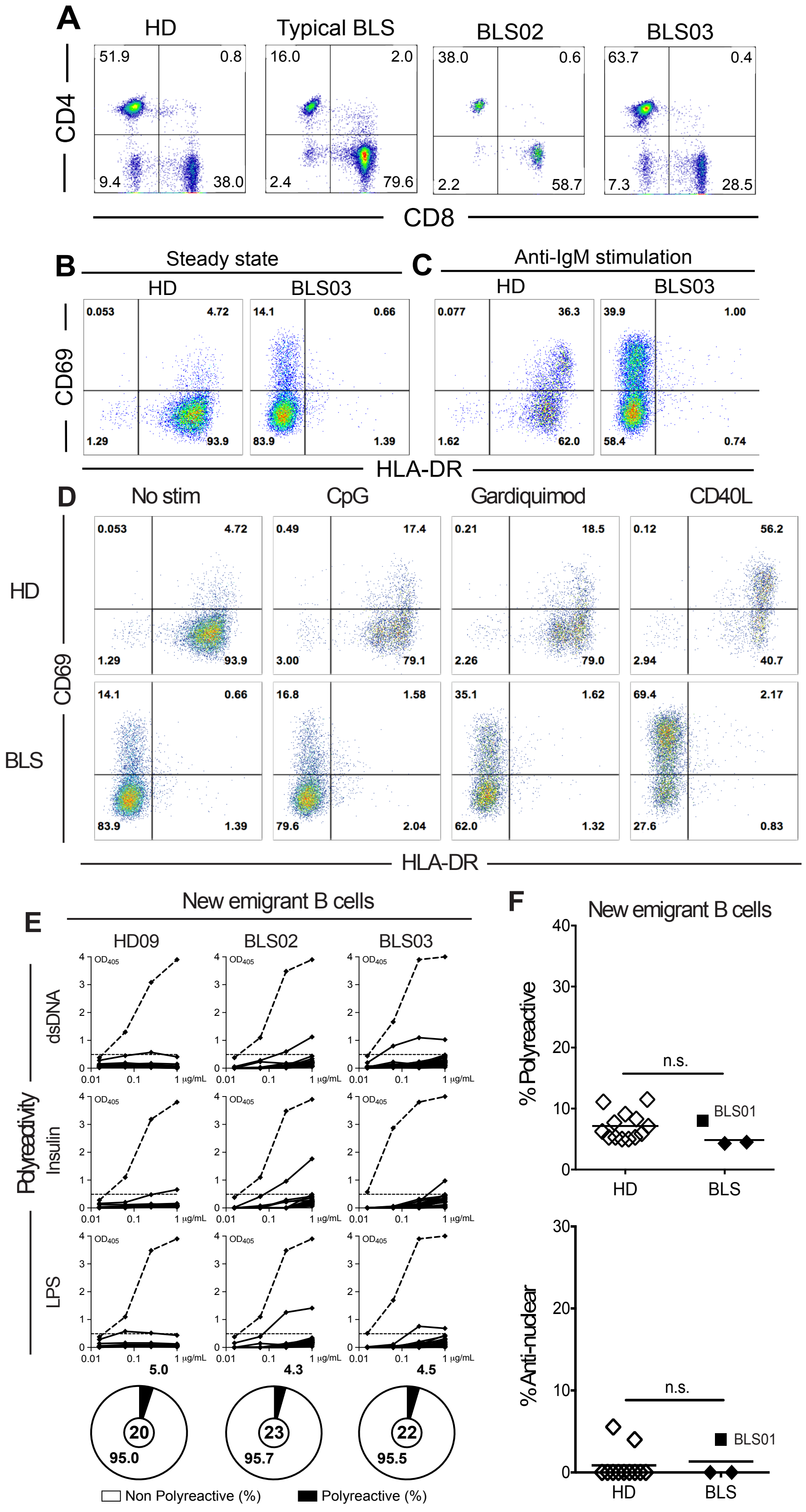
A
HD
Typical BLS
BLS02
BLS03
CD4
CD8
51.9
0.8
9.4
38.0
16.0
2.0
2.4
79.6
38.0
0.6
2.2
58.7
63.7
0.4
7.3
28.5
B
Steady state
HD
BLS03
C
Anti-IgM stimulation
HD
BLS03
CD69
HLA-DR
0.053
4.72
1.29
93.9
14.1
0.66
83.9
1.39
0.077
36.3
1.62
62.0
39.9
1.00
58.4
0.74
D
No stim
CpG
Gardiquimod
CD40L
HD
BLS
CD69
HLA-DR
0.053
4.72
1.29
93.9
0.49
17.4
3.00
79.1
0.21
18.5
2.26
79.0
0.12
56.2
2.94
40.7
14.1
0.66
83.9
1.39
16.8
1.58
79.6
2.04
35.1
1.62
62.0
1.32
69.4
2.17
27.6
0.83
E
New emigrant B cells
HD09
BLS02
BLS03
Polyreactivity
dsDNA
Insulin
LPS
OD405
0.01
0.1
1 µg/mL
5.0
4.3
4.5
20
23
22
95.0
95.7
95.5
Non Polyreactive (%)
Polyreactive (%)
F
New emigrant B cells
% Polyreactive
n.s.
BLS01
HD
BLS
% Anti-nuclear
n.s.
BLS01
HD
BLS

Figure S7. Proper central B cell tolerance in atypical BLS patients who fail to express MHC class II on their B cells. (**A**) Representative CD4 vs. CD8 flow cytometry analysis (gated on $CD3^+$ T cells) of a healthy donor (HD), a typical BLS patient and the two atypical BLS patients enrolled for this study. (**B**) Representative flow analysis of activation marker CD69 and HLA-DR in purified B cells at steady state and (**C**) after 48 hours of anti-IgM stimulation. (**D**) Flow cytometry analysis of activation marker CD69 and HLA-DR in purified B cells that were non-stimulated (No stim) or stimulated with TLR9 ligand CpG, TLR7 ligand gardiquimod, or CD40 ligand (CD40L). Top panel shows HD B cells and bottom panel shows B cells from a BLS patient. (**E**) Polyreactivity of recombinant antibodies cloned from single new emigrant/transitional B cells from the two atypical BLS patients were assessed by ELISAs and compared with a representative healthy donor (HD). Dotted lines show the ED38 positive control. Horizontal lines show the cut-off OD_{405} for positive reactivity. For each subject, the frequency of non-reactive (open area) and reactive (filled area) clones is summarized in pie charts, with the total number of clones tested indicated in the centers. (**F**) Summary of polyreactivity (top) and anti-nuclear reactivity (bottom) in new emigrant/transitional B cells. Each open diamond represents a healthy individual and each filled diamond represents an atypical BLS patient from this study. The filled square represents a previously reported classical BLS patient (Herve *et al.*, 2007).

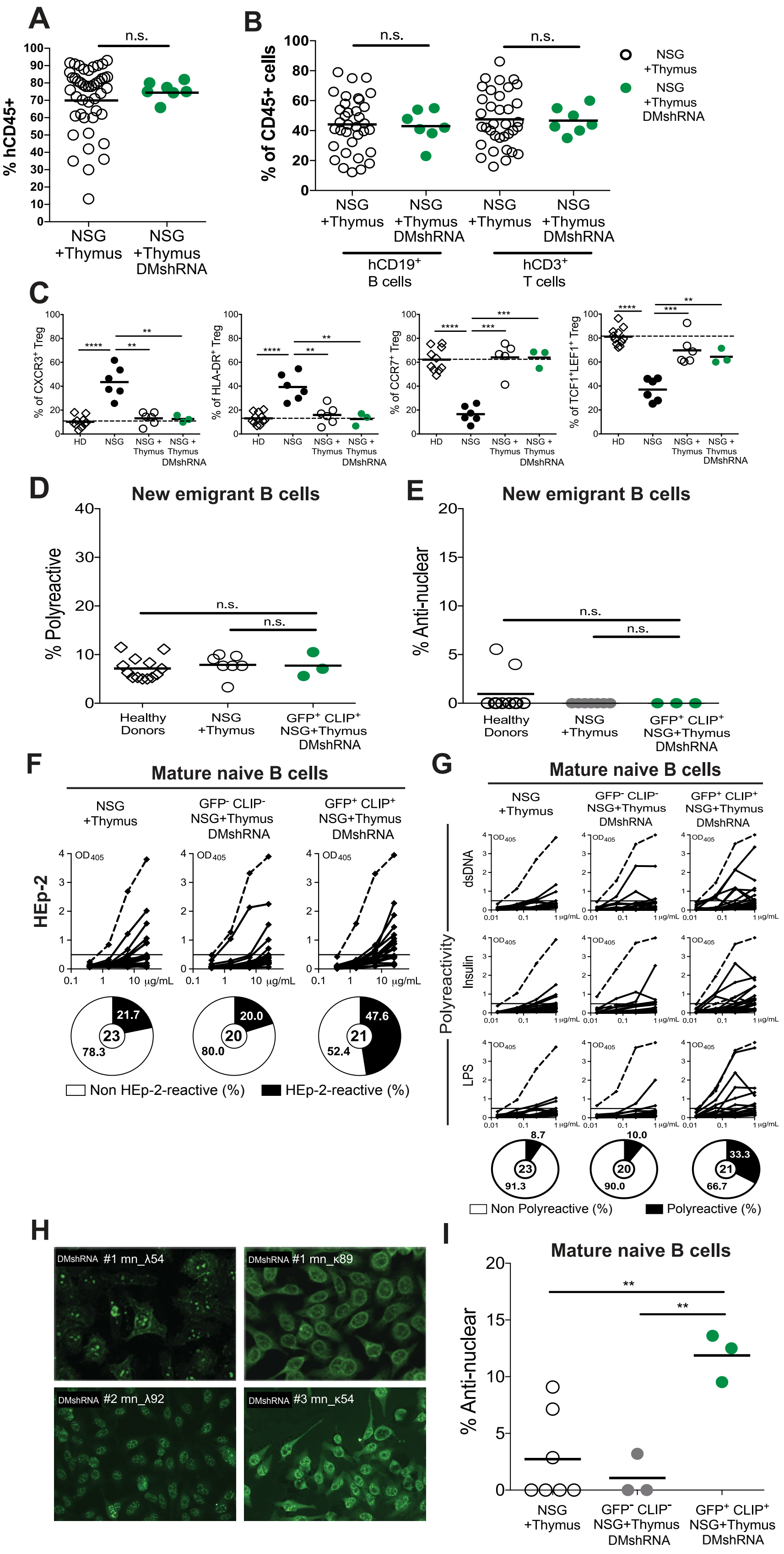

A
% hCD45+
NSG +Thymus
NSG +Thymus DMshRNA
n.s.
B
% of CD45+ cells
NSG +Thymus
NSG +Thymus DMshRNA
hCD19+ B cells
hCD3+ T cells
n.s.
C
% of CXCR3+ Treg
% of HLA-DR+ Treg
% of CCR7+ Treg
% of TCF1+LEF1+ Treg
HD
NSG
NSG + Thymus
NSG + Thymus DMshRNA
D
New emigrant B cells
% Polyreactive
Healthy Donors
NSG +Thymus
GFP+ CLIP+ NSG+Thymus DMshRNA
E
New emigrant B cells
% Anti-nuclear
F
Mature naive B cells
HEp-2
GFP- CLIP- NSG+Thymus DMshRNA
OD405
μg/mL
21.7
23
78.3
20.0
20
80.0
47.6
21
52.4
Non HEp-2-reactive (%)
HEp-2-reactive (%)
G
Mature naive B cells
Polyreactivity
dsDNA
Insulin
LPS
8.7
91.3
10.0
90.0
33.3
66.7
Non Polyreactive (%)
Polyreactive (%)
H
DMshRNA #1 mn_λ54
DMshRNA #1 mn_κ89
DMshRNA #2 mn_λ92
DMshRNA #3 mn_κ54
I
Mature naive B cells
% Anti-nuclear

Figure S8. HLA-DM knockdown in NSG+Thymus humanized mice does not affect human lymphocyte engraftment, Treg phenotype, Treg suppression or central B cell tolerance, but results in the accumulation of autoreactive mature naïve B cells. (**A**) Summary of human $CD45^+$ leukocyte engraftment in the blood of unmanipulated NSG+Thymus humanized mice or those engrafted with HSCs transduced with GFP-tagged lentivirus expressing an *HLADM* specific shRNA. (**B**) Summary of human $CD19^+$ B cells and $CD3^+$ T cells as % of $hCD45^+$ cells in the blood of indicated humanized mice. (**C**) Summary of flow cytometry analysis of CXCR3, HLA-DR, CCR7, TCF1, LEF-1 Treg markers described in Figure 4 reveals that Tregs from *HLADM* shRNA NSG+Thymus humanized mice express these molecules at similar levels as those in HD and unmanipulated NSG+Thymus humanized mice. $^{**}P < 0.01$; $^{***}P < 0.001$; $^{****}P < 0.0001$ (Mann-Whitney U test). (**D**) Polyreactivity and anti-nuclear reactivity. (**E**) Summaries of GFP^+CLIP^+ new emigrant/transitional B cells from *HLADM* shRNA NSG+Thymus humanized mice compared to those of unmanipulated NSG+Thymus humanized mice and healthy donors. HEp-2 reactivity (**F)** and polyreactivity (**G**) of recombinant antibodies cloned from single mature naïve B cells unaltered (GFP^-) or HLA-DM-silenced (GFP^+CLIP^+) were assessed by ELISAs and compared with those from a representative NSG+Thymus humanized mouse. (**H**) Indirect immunofluorescence imaging of HEp2 cell lysates by recombinant antibodies cloned from mature naïve B cells of *HLADM* shRNA NSG+Thymus humanized mice reveal various patterns of nuclear and cytoplasmic staining. (**I**) Summary of the frequencies of anti-nuclear clones in the indicated B cell group. $^{**}P < 0.01$, $^{***}P < 0.001$, $^{****}P < 0.0001$ (Kruskal-Wallis test).

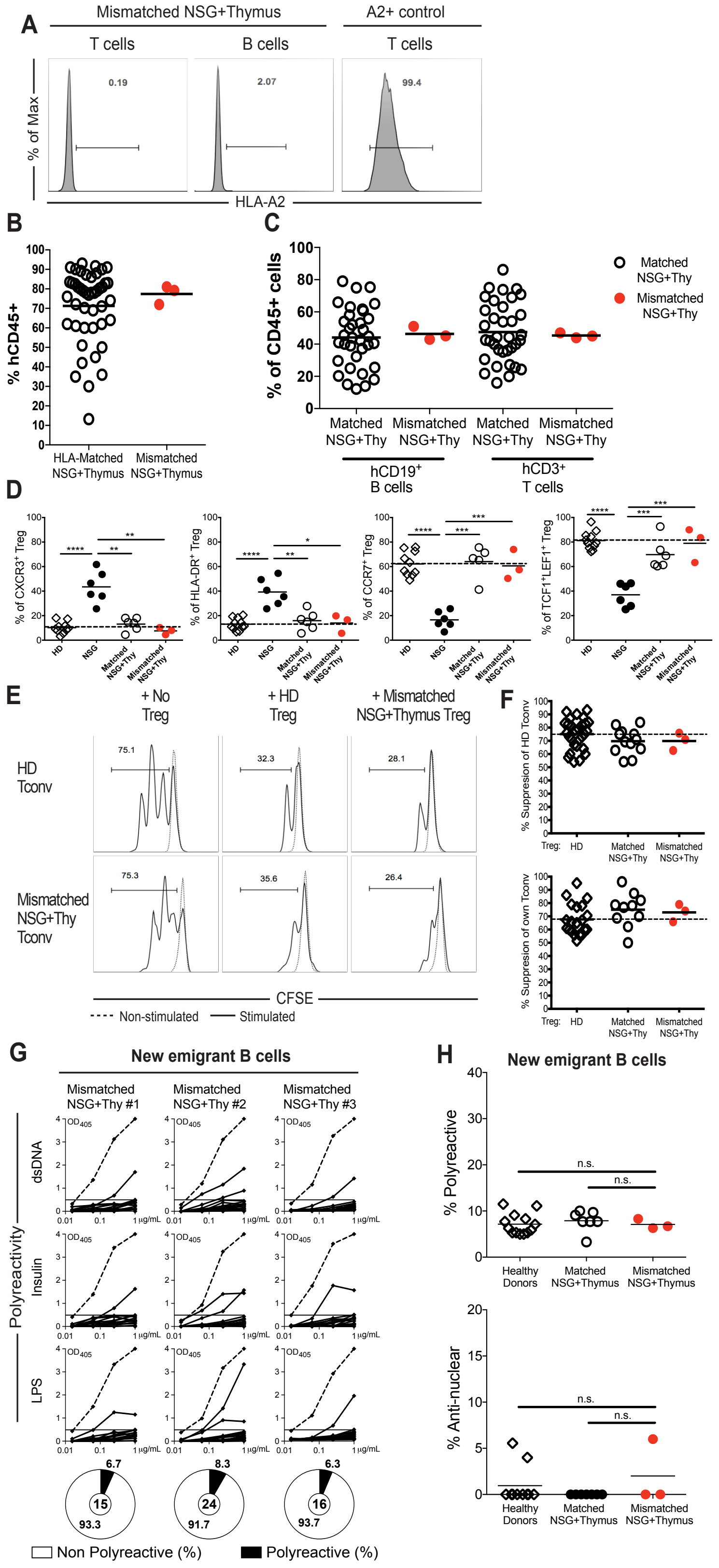

A
Mismatched NSG+Thymus
A2+ control
T cells
B cells
T cells
0.19
2.07
99.4
% of Max
HLA-A2
B
% hCD45+
HLA-Matched NSG+Thymus
Mismatched NSG+Thymus
C
% of CD45+ cells
Matched NSG+Thy
Mismatched NSG+Thy
hCD19+ B cells
hCD3+ T cells
D
% of CXCR3+ Treg
% of HLA-DR+ Treg
% of CCR7+ Treg
% of TCF1+LEF1+ Treg
HD
NSG
Matched NSG+Thy
Mismatched NSG+Thy
E
+ No Treg
+ HD Treg
+ Mismatched NSG+Thymus Treg
HD Tconv
Mismatched NSG+Thy Tconv
75.1
32.3
28.1
75.3
35.6
26.4
CFSE
Non-stimulated
Stimulated
F
% Suppresion of HD Tconv
% Suppresion of own Tconv
Treg:
HD
Matched NSG+Thy
Mismatched NSG+Thy
G
New emigrant B cells
Mismatched NSG+Thy #1
Mismatched NSG+Thy #2
Mismatched NSG+Thy #3
Polyreactivity
dsDNA
Insulin
LPS
OD405
μg/mL
6.7
8.3
6.3
15
24
16
93.3
91.7
93.7
Non Polyreactive (%)
Polyreactive (%)
H
New emigrant B cells
% Polyreactive
% Anti-nuclear
n.s.
Healthy Donors
Matched NSG+Thymus
Mismatched NSG+Thymus

Figure S9. HLA-mismatched NSG+Thymus humanized mice display normal human lymphocyte engraftment, Treg phenotype, Treg suppressive function, and functional central B cell tolerance. (**A**) Representative flow cytometry analysis of HLA-A2 expression shows that both peripheral T cells and B cells originated from the engrafted HSCs (HLA-A2$^-$). A positive control for HLA-A2 expression is shown on the right. Summary of human CD45$^+$ leukocyte engraftment (**B**) and human CD19$^+$ B cells and CD3$^+$ T cells as % of hCD45$^+$ (**C**) in the blood of HLA-matched and HLA-mismatched NSG+Thymus humanized mice. (**D**) Summary of flow cytometry analysis of CXCR3, HLA-DR, CCR7, TCF1, LEF-1 Treg markers reveals that Tregs from HLA-mismatched NSG+Thymus humanized mice express these molecules at similar levels as those in HD and HLA-matched NSG+Thymus humanized mice. *P < 0.05; **P < 0.01; ***P < 0.001; ****P < 0.0001 (Kruskal-Wallis test). (**E**) Representative flow cytometry analysis of Treg-mediated *in vitro* suppression of autologous and heterologous CFSE-labeled conventional T cells (Tconv) on day 3.5. Dashed line shows non-stimulated Tconv. Tregs from HLA-mismatched NSG+Thymus humanized mice were able to suppress the proliferation of both autologous and healthy donor T cells. (**F**) Summaries of suppressive capacity of Tregs from healthy donors (HD), HLA-matched and mismatched NSG+Thymus mice in heterologous (top; with HD Tconv) and autologous (bottom; with humanized mouse Tconv) settings. (**G**) Polyreactivity of recombinant antibodies cloned from new emigrant/transitional B cells from three HLA-mismatched NSG+Thymus humanized mice were assessed by ELISAs. (**H**) Summaries of polyreactivity (top) and anti-nuclear reactivity (bottom) of new emigrant/transitional B cells from HLA-mismatched NSG+Thymus humanized mice compared to those of HLA-matched NSG+Thymus humanized mice and HD.

Key resources

REAGENT or RESOURCE	SOURCE	IDENTIFIER
**Antibodies**		
Pacific Blue anti-human CD19 (Clone H1B19)	Biolegend	Cat# 302224
FITC anti-human IgM (Clone G20-127)	BD Biosciences	Cat# 555782
PerCP-Cy5.5 anti-human IgM (Clone MHM-88)	Biolegend	Cat# 314511
PE-Cy7 anti-human CD10 (Clone HI10a)	Biolegend	Cat# 312214
APC anti-human CD21 (Clone B-ly4)	BD Biosciences	Cat# 559867
PerCP-Cy5.5 anti-human CD27 (Clone M-T271)	Biolegend	Cat# 356408
PE anti-HLA-DM (clone MaP.DM1)	Biolegend	Cat# 358003
APC anti-human CLIP (Clone REA296)	Miltenyi Biotec	Cat# 130-104-421
eVolve 605 anti-human CD3 (Clone OKT3)	eBioscience	Cat# 83-0037-42
APC-Cy7 anti-human CD4 (Clone OKT4)	Biolegend	Cat# 317416
PE-Cy7 anti-human CD8 (Clone HIT8)	Biolegend	Cat# 300913
PE anti-human CD25 (Clone BC96)	Biolegend	Cat# 302606
Alexa Fluor 647 anti-human CD127 (Clone A09D5)	Biolegend	Cat# 351318
Alex Flour 488 anti-human FOXP3 (Clone PCH101)	eBioscience	Cat# 53-4776-41
PE anti-human CTLA-4 (Clone BNI3)	BD Biosciences	Cat# 560939
Alexa Fluor 647 anti-human HELIOS (Clone 22F6)	Biolegend	Cat# 137218
Alexa Fluor 700 anti-human CXCR3 (G025H7)	Biolegend	Cat# 353741
Alexa Fluor 647 anti-human ICOS (Clone C39A.4A)	Biolegend	Cat# 313510
Alexa Fluor 700 anti-human HLA-DR (Clone L243)	Biolegend	Cat# 307626
Alexa Fluor 647 anti-human CCR7 (Clone G043H7)	Biolegend	Cat# 353218
Alex Flour 617 anti-human TCF1	Cell Signaling Tech	Cat# 6709S
PE anti-human LEF1	Cell Signaling Tech	Cat# 14440S
Anti-human CD25 antibody (IgG1, Clone IL2R.1)	LSBio	Cat# LS-C87934
Anti-human CD25 antibody (IgG2a)	LSBio	Cat# LS-B7396
Anti-human CD2 antibody (Clone T6.3)	LSBio	Cat# LS-C140259
**Biological Samples**		
Healthy donor blood samples	Yale School of Medicine	N/A
Patient blood samples	Catharina Schutz	BLS02 and BLS03
**Chemicals, Peptides, and Recombinant Proteins**		
Insulin	Sigma-Aldrich	Cat# I2543-25MG
Lipopolysaccharides (LPS)	Sigma-Aldrich	Cat# L6529-1MG
Deoxyribonucleic acid sodium salt (dsDNA)	Sigma-Aldrich	Cat# D3664-5X2MG
Human SCF	R&D Systems	Cat# 255-SC-050
Human Flt3-L	R&D Systems	Cat# 308-FK-025
Human IL-3	R&D Systems	Cat# 203-IL-050
**Critical Commercial Assays**		
QUANTA Lite HEp-2 ELISA	Inova Diagnostics	Cat# 708750
ANA (HEp-2) slides for immunofluorescent imaging	BIon Enterprises, Ltd	Cat# ANK-120
Treg suppression inspector beads	Miltenyi Biotec	Cat# 120-092-909
CD34 MicroBead kit	Miltenyi Biotec	Cat# 130-100-453
CD19 MicroBead kit	Miltenyi Biotec	Cat# 130-050-301

Deposited Data		
ImmunoSEQ data	Adaptive Biotech	URL:https://clients.adaptivebiotech.com/pub/chen-2021-jci. DOI: 10.21417/JWC2021JCI
RNA-seq data	NCBI GEO	GSE #186881
**Experimental Models: Cell Lines**		
Cell line: HEK293	ATCC	Cat# CRL-1573
**Experimental Models: Organisms/Strains**		
NOD/scid/IL-2Rgamma^{null} (NSG) mice	Jackson Laboratory	Stock# 005557
**Recombinant DNA**		
pSUPER vector	(Cantaert et al., 2015)	N/A
pTrip-Ubi-GFP vector	(Cantaert et al., 2015)	N/A

Materials availability

Further information and requests for resources and reagents should be directed to and will be fulfilled by the Lead Contact, Eric Meffre (eric.meffre@yale.edu).

Table S1 (see Excel file). Repertoire and reactivity of antibodies cloned from new emigrant/transitional and mature naïve B cells from humanized mice and patients

Table S2. List of TCR V$_\beta$ genes that were differentially expressed in batch-sorted Tregs from NSG vs. NSG+Thymus humanized mice

Gene symbol	IMGT nomenclature	Log2FC	Adjusted P value
TCRBV12S2	TRBV10-3	1.41	4.71E-03
TCRBV7S3	TRBV4-2	1.33	5.00E-04
TCRBV15S1	TRBV24	1.26	1.27E-05
TCRBV2S1	TRBV20	-1.02	1.68E-04

Table S3. Summary of BLS patient characteristics

Patient #	Study	Gene mutation	Gender	Age at diagnosis	Symptoms
BLS01	Herve et al.(2007)	*CIITA*	M	3	Pneumonocystis carinii pneumonia hypogammaglobulinemia
BLS02	Current	*RFXAP*	F	15	Type I diabetes; Klebsiella pneumonia; Candida keratitis
BLS03	Current	*RFXANK*	F	2	Failure to thrive; recurrent pneumonia; osteopenia

Table S4. HLA Class II and Class I alleles of HSC and thymic graft donors used to generate three sets of HLA-mismatched NSG+Thymus humanized mice

	HLA Locus	DRB1	DQB1	DPB1	A	B	C
#1	Thymus	04:04 / 15:01	03:02 / 06:02	04:01P / 04:02P	A2 / A2	B60 / B48	Cw10 / Cw8
	HSCs	13:01 / 16:01	05:02 / 06:03	02:01P / 04:02P	A1 / A11	B35 / B39	Cw4 / Cw12
#2	Thymus	08:01 / 13:02	04:02 / 06:04	02:01P / 03:01P	A2 / A1	B63 / B39	Cw7 / Cw12
	HSCs	01:01 / 07:01	02:02 / 05:01	04:01P / 04:02P	A3 / A31	B35 / B37	Cw4 / Cw6
#3	Thymus	04:01 / 15:01	03:02 / 06:02	04:01P / 11:01	A2 / A26	B8 / B62	Cw9 / Cw10
	HSCs	07:01 / 12:02	02:02 / 03:01	03:01P / 14:01	A3 / A11	B27 / B50	Cw6 / Cw12
